# Overexpression of *MdbHLH104* gene enhances the tolerance to iron deficiency in apple

**DOI:** 10.1111/pbi.12526

**Published:** 2016-01-23

**Authors:** Qiang Zhao, Yi‐Ran Ren, Qing‐Jie Wang, Yu‐Xin Yao, Chun‐Xiang You, Yu‐Jin Hao

**Affiliations:** ^1^ National Key Laboratory of Crop BiologyNational Research Center for Apple Engineering and TechnologyCollege of Horticulture Science and EngineeringShandong Agricultural UniversityTai‐AnShandongChina

**Keywords:** apple, IVc subgroup bHLH transcription factor, plasma membrane H^+^‐ATPase, iron deficiency, iron uptake

## Abstract

Fe deficiency is a widespread nutritional disorder in plants. The basic helix‐loop‐helix (bHLH) transcription factors (TFs), especially Ib subgroup bHLH TFs which are involved in iron uptake, have been identified. In this study, an IVc subgroup bHLH TF MdbHLH104 was identified and characterized as a key component in the response to Fe deficiency in apple. The overexpression of the *MdbHLH104* gene noticeably increased the H^+^‐ATPase activity under iron limitation conditions and the tolerance to Fe deficiency in transgenic apple plants and calli. Further investigation showed that MdbHLH104 proteins bonded directly to the promoter of the *MdAHA8* gene, thereby positively regulating its expression, the plasma membrane (PM) H^+^‐ATPase activity and Fe uptake. Similarly, MdbHLH104 directly modulated the expression of three Fe‐responsive bHLH genes, *MdbHLH38*,* MdbHLH39* and *MdPYE*. In addition, MdbHLH104 interacted with 5 other IVc subgroup bHLH proteins to coregulate the expression of the *MdAHA8* gene, the activity of PM H^+^‐ATPase and the content of Fe in apple calli. Therefore, MdbHLH104 acts together with other apple bHLH TFs to regulate Fe uptake by modulating the expression of the *MdAHA8* gene and the activity of PM H^+^‐ATPase in apple.

## Introduction

As an essential mineral element for plants, iron is required for DNA synthesis, photosynthesis, nitrogen fixation, hormone synthesis and electron transport in the respiratory chain (Briat and Lobréaux, 1997). Although the total Fe content in earth generally satisfies plants' requirement, its availability is very low due to the unsuitable pH environments and low solubility in calcareous soils and anaerobic conditions, and approximately 30% of the world's soils are considered Fe limiting for plant growth (Korcak, 1988). Therefore, Fe deficiency is one of the major factors limiting plant growth and development (Guerinot and Yi, 1994). Thus, to maintain appropriate amounts of iron, plants have developed a number of sophisticated mechanisms to acclimate themselves to the surrounding conditions, including Fe limitation.

The need for the efficient acquisition of iron from soil has resulted in the evolution of two phylogenetically distinct uptake strategies, that is strategy I in dicotyledonous plants and strategy II in graminaceous monocots. Strategy I plants uptake Fe in a three‐step process: the solubilization of Fe^3+^ complexes through rhizosphere acidification, the reduction of ferric (Fe^3+^) into ferrous (Fe^2+^) and lastly, the uptake of the ferrous into root cells (Marschner, 1995; Römheld and Marschner, 1986; Santi and Schmidt, 2009). In contrast, strategy II plants synthesize mugineic acids in root and chelate iron to form Fe^3+^–MA complexes, which are transported into the cells of roots by yellow stripe transporters (Marschner and Römheld, 1986; Römheld and Marschner, 1986).

In strategy I plants, the transformation of Fe^3+^ to Fe^2+^ depends on ferric oxidoreductase ferric reductase oxidase 2 (FRO2), whereas the uptake of Fe^2+^ into root cells depends on iron transporter iron‐regulated transporter 1 (IRT1). Both *FRO2* and *IRT1* genes are induced by Fe deficiency. It has been found that bHLH transcriptional factors (TFs) are involved in the regulation of Fe acquisition and homeostasis. In *Arabidopsis*, genes of bHLH subgroups Ib and IVc are induced by iron‐deficient conditions (Li *et al*., 2006). The Ib subgroup bHLH TFs characterized with a function in Fe deficiency are FER in tomato and its homolog FIT (FER‐like iron deficiency‐induced transcription factor) in *Arabidopsis* (Ling *et al*., 2002; Yuan *et al*., 2005). FIT directly binds to the promoters of *FRO2* and *IRT1* and up‐regulates their expression under Fe deficiency (Colangelo and Guerinot, 2004).

Prior to the reduction of Fe^3+^ to Fe^2+^, Fe^3+^ complexes must be solubilized through rhizosphere acidification (Marschner, 1995). In dicotyledonous plants, the plasma membrane (PM) H^+^‐ATPase (EC 3.6.1.35) is responsible for the proton extrusion out of cells and the formation of rhizosphere acidification, which has a huge effect on the soluble of Fe in the vicinity of the roots (Dell'Orto *et al*., 2000; Schmidt, 2003). In addition, the action of PM H^+^‐ATPase generates an electrochemical gradient, which constitutes a driving force for the transport of mineral nutrients, toxic ions, solutes and metabolites across the PM. Therefore, PM H^+^‐ATPase plays a crucial role in plants' responses to various environmental factors such as saline stress, low solution pH, nutrient supply and Fe deficiency (Niu *et al*., 1993; Schubert and Yan, 1997; Yan *et al.,* 1998; Dell'Orto *et al*., 2000; Palmgren, 2001).

In *Arabidopsis*, PM H^+^‐ATPases are encoded by 11 *AHA* genes, which are induced by various environmental stimuli. Among them, the expressions of *AHA2*,* AHA3*,* AHA4* and *AHA7* are up‐regulated by Fe deficiency. In response to the absence of iron, *AHA2* is responsible for the major acidification activity, whereas *AHA7* may regulate root hair formation (Santi and Schmidt, 2009). Compared with the direct regulation of *FRO2* and *IRT1* by Ib bHLH TF FIT, it is largely unknown whether and how the PM H^+^‐ATPase gene is regulated by bHLH TF. Although *AHA2* expression is up‐regulated in *FIT* overexpression plants than *fit‐3* mutant in response to iron deficiency (Ivanov *et al*., 2012; Long *et al*., 2010), several evidences show that it appears not to be directly controlled by FIT, suggesting a different induction pathway for *AHA2* gene, compared with that for *IRT1* and *FRO2* genes (Ivanov *et al*., 2012; Santi and Schmidt, 2009).

In addition to those of the Ib subgroup, IVc subgroup bHLHs influence the Fe chelate reductase activity and the acidification of rhizospheres to regulate plant growth and development under Fe‐deficient conditions. Among them, PYE and IAA‐Leu Resistant3 (ILR3, also named as bHLH105) target metal (or iron) homeostasis genes, which are involved in intracellular and long‐distance metal (or iron) transport (Rampey *et al*., 2006; Selote *et al*., 2015). Meanwhile, PYE regulates the acidification of rhizospheres under Fe‐deficient conditions (Long *et al*., 2010; Selote *et al*., 2015). Most recently, it is found in *Arabidopsis* that the mutations to IVc subgroup bHLH genes *AtbHLH104* and *AtbHLH105* greatly reduce the tolerance to Fe deficiency, whereas their overexpressions improve the tolerance and lead to an accumulation of excess Fe under soil‐grown conditions. *AtbHLH104* also regulates the acidification of rhizospheres under Fe‐deficient conditions (Zhang *et al*., 2015). In chrysanthemum, *CmbHLH1*, which is highly similar to *AtbHLH105*, regulates Fe uptake via mediating the acidification of the rhizosphere by enhancing the transcription of the H^+^‐ATPase‐encoding gene *CmHA* under iron‐shortage conditions (Zhao *et al*., 2014).

In this study, a Fe‐responsive bHLH TF gene *MdbHLH104* was isolated from the apple. It was identified to encode an IVc bHLH subgroup member and was induced by Fe deficiency. After being genetically transformed into apple plant and calli, MdbHLH104 was characterized by a crucial function in Fe acquisition and the tolerance to Fe deficiency by directly binding to the promoter regions of the *MdAHA8*,* MdbHLH38*,* MdbHLH39* and *MdPYE* genes, thereby modulating PM H^+^‐ATPase activity. Finally, the potential utilization of *MdbHLH104* in the genetic improvement of fruit tree tolerance to iron deficiency is discussed.

## Results

### bHLH transcription factor MdbHLH104 is involved in responding to iron deficiency and promotes iron accumulation

**Figure 1:**
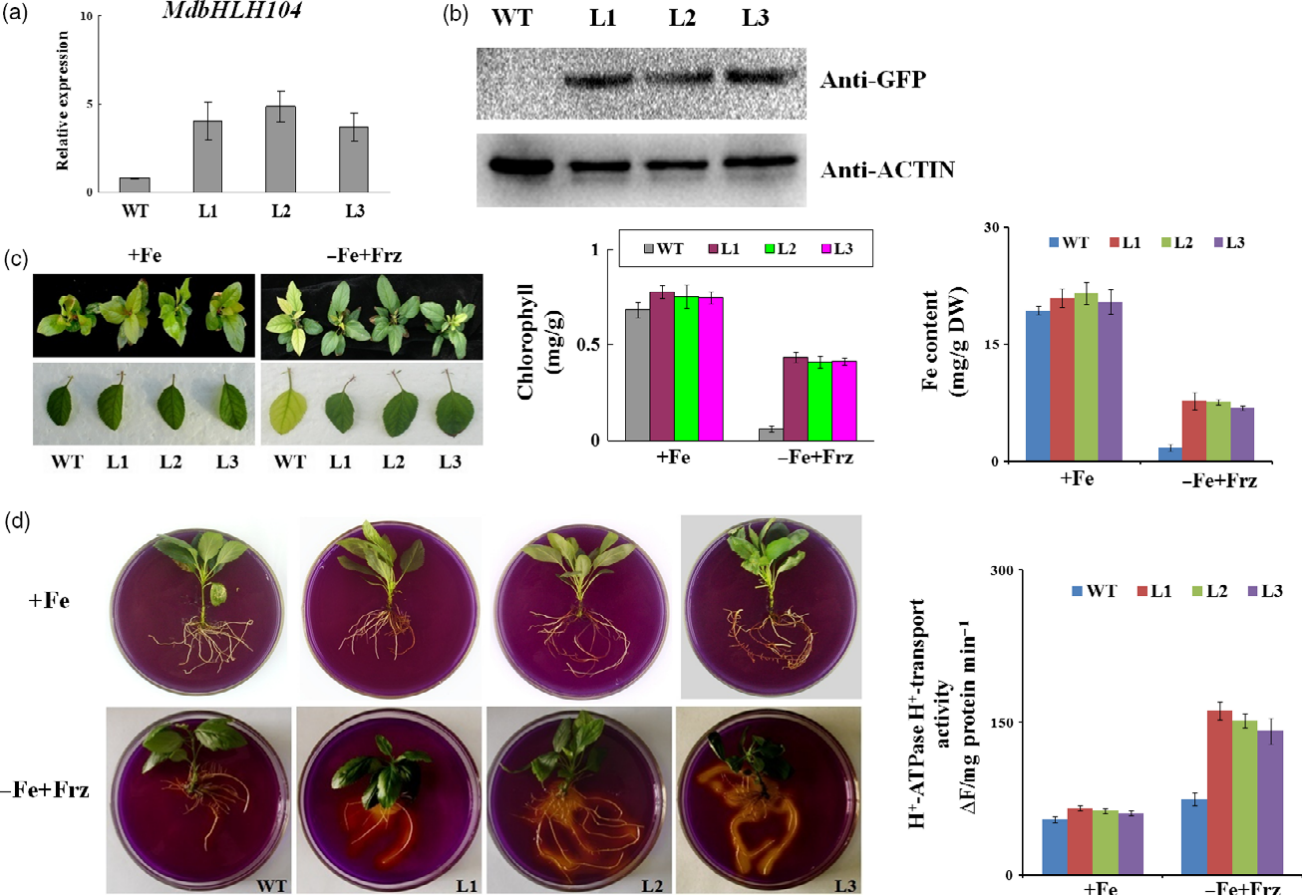
The phenotype of *MdbHLH104* transgenic apple plantlets under Fe‐sufficient and deficient conditions. (a) Expression level of the *MdbHLH104* gene in the transgenic apple lines and the wild‐type (empty vector WT control). (b) The level of the MdbHLH104‐GFP fusion protein in *35S::MdbHLH104‐GFP* transgenic apple lines, as determined by immunoblot analysis using an anti‐GFP antibody. The anti‐actin antibody was used as loading control. (c) The appearance, total chlorophyll contents and iron contents of *35S::MdbHLH104‐GFP* transgenic apple lines and the WT control grown for 1 month on Fe‐sufficient (+Fe) or Fe‐deficient (−Fe + Frz) media. The data represent the means ± SD of three independent experiments. DW: dry weight. (d) The rhizosphere acidification and PM H^+^‐ATPase activity of wild‐type and *35S::MdbHLH104‐GFP* transgenic apple lines grown for 7 days on Fe‐sufficient (+Fe) or Fe‐deficient (−Fe + Frz) media. The yellow colour around the roots stained with bromocresol purple indicates rhizosphere acidification, and the plasma membrane vesicles were isolated for PM H^+^‐ATPase activity analysis. The data represent the means ± SD of three independent experiments.

BlastX search and phylogenetic tree analysis showed that there are 6 IVc subgroup bHLH TFs in apple (Figure S1a,b). Among them, the expression of the *MdbHLH104* gene was noticeably induced by Fe deficiency (Figure S1c). It is also highly expressed in root (Figure S1d). To characterize its function, an expression vector *35S::MdbHLH104‐GFP* was constructed and transformed into apple with an *Agrobacterium*‐mediated method. As a result, five independent transgenic apple lines were obtained (Figure S2). Three lines, L1, L2 and L3, were chosen for further investigation, whereas the wild‐type (WT) apple was used as a control. Expression analysis and an immunoblot assay with an anti‐GFP antibody showed that all three transgenic apple lines generated many more *MdbHLH104* transcripts and produced MdbHLH104‐GFP fusion proteins (Figures 1a,b and S2d), indicating that *MdbHLH104* was overly expressed in apple.

To examine whether MdbHLH104 protein plays a role in response to iron starvation, three transgenic apple lines and the WT control were allowed to grow for 20 days under Fe‐sufficient conditions and then shifted to Fe‐deficient conditions for another 30 days. The results showed that the both transgenic and WT apple plantlets grew normally under Fe‐sufficient conditions. After being treated with Fe starvation, the WT control exhibited much more severe chlorosis in appearance (Figure 1c), which was also indicated as low chlorophyll contents in the unfolding young leaves than in three transgenic lines (Figure 1c). In contrast, the three transgenic lines showed much less chlorosis than the control (Figure 1c). Furthermore, the iron content was measured in the unfolding young leaves. The result indicated that three *35S::MdbHLH104‐GFP* transgenic lines accumulated much higher iron than the WT control under iron‐deficient conditions (Figure 1c). These results indicated that the overexpression of *MdbHLH104* confers remarkably increased tolerance to Fe deficiency in transgenic apple plantlets.

Plant root responds to iron deprivation by pumping out protons into the apoplast, which lowers the rhizosphere pH and solubilizes iron, thus increasing iron availability (Yi *et al*., 1994). To test whether MdbHLH104 influences the rhizosphere pH in response to iron deprivation, the transgenic and WT apple plantlets grown under normal conditions were treated for 7 days on a Fe‐deficient medium. Subsequently, they were shifted to a medium containing the pH indicator bromocresol purple for staining. The result showed that transgenic lines exhibited more obvious rhizosphere acidification, as indicated by the yellow colour of the medium around the roots, than the WT control under Fe‐deficient conditions, and no phenotypic differences between the transgenic lines and WT control were revealed under Fe‐sufficient conditions. Furthermore, the transport activity of PM H^+^‐ATPase was determined. The result indicated that the three transgenic lines exhibited a notably increased ATPase activity relative to the WT control under Fe‐deficient conditions and no changes under Fe‐sufficient condition (Figure 1e). These findings demonstrated that MdbHLH104 overexpression leads to an increased acidification of the rhizosphere in response to iron deficiency.

### MdbHLH104 binds to the promoter of *MdAHA8* and activates its transcription

**Figure 2:**
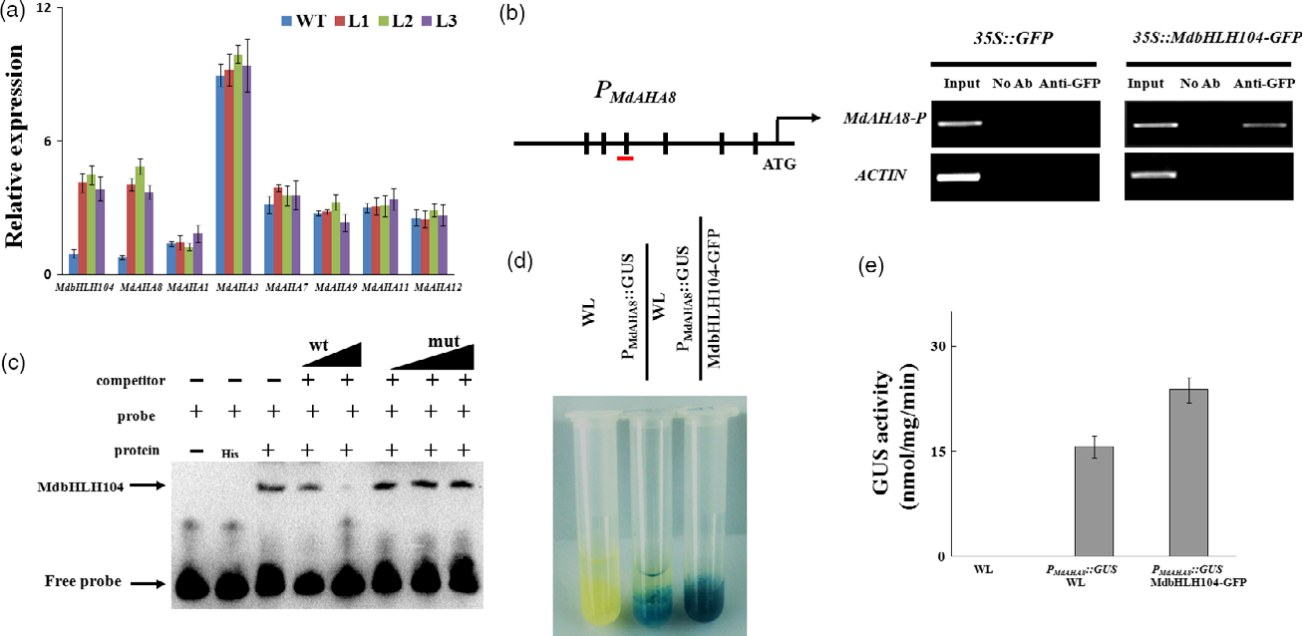
MdbHLH104 directly activates the expression of *MdAHA8* gene. (a) qRT‐PCR assays for *MdbHLH104* and *MdAHA* genes in transgenic apple lines. (b) Illustration of the *MdAHA8* promoter region indicating the presence of E‐box DNA motifs. Transverse lines show the positions of primers used in the ChIP‐PCR experiment. ChIP assays were performed using the *35S::GFP* and *35S::MdbHLH104‐GFP* apple calli. A region containing E‐box in the actin promoter is negative control. (c) MdbHLH104 binds to the E‐box motifs present in the *MdAHA8* promoter *in vitro*, as indicated by an EMSA method. The *MdAHA8* promoter fragment containing the E‐box motifs was incubated with His‐MdbHLH104 protein. Competition for MdbHLH104 binding was performed with 50× and 100× unlabelled probes (wt) or G‐box‐mutated probes (mut). His was used as the control. Mut indicates mutated probes. ‘+’ indicates presence, and ‘−’ indicates absence. (d) and (e) GUS staining assay and activity analysis of *MdAHA8* expression promoter using *P*_*M*__*d*_ _*AHA*__*8*_ *::GUS* and *35S::MdbHLH104‐GFP+* *P*_*M*__*d*_ _*AHA*__*8*_ *::GUS* transgenic apple calli. GUS activity was measured using a 4‐methylumbelliferyl‐d‐glucuronide assay. The data represent the means ± SD of three independent experiments.

Among 11 *Arabidopsis* AHAs, AHA1 and AHA2 are two major PM H^+^‐ATPases responsible for rhizosphere acidification (Santi and Schmidt, 2009). Correspondingly, there are 18 *MdAHA* genes in the apple genome (http://genomics.research.iasma.it/). The phylogenetic tree demonstrated that 7 MdAHAs are close to AHA1 and AHA2 (Figure S3a). RT‐PCR analysis showed that among them, only *MdAHA8* was noticeably up‐regulated in the transgenic lines, compared with the WT control under Fe‐deficient conditions (Figure 2a).

The bHLH transcription factors have been reported to be associated with the E‐box (5′‐CANNTG‐3′) or G‐box (5′‐CACGTG‐3′) cis element in the promoters of their target genes (Fisher and Goding, 1992). To elucidate how *MdAHA8* is regulated by MdbHLH104, its promoter region was searched for putative *cis* elements that are recognized by MdbHLH104. As a result, 6 E‐box elements (CANNTG), that is P1 to P6, were found (Figures 2b and S3b). To verify whether MdbHLH104 binds to those elements, a chromatin immunoprecipitation PCR (ChIP‐PCR) assay was conducted with an anti‐GFP antibody and six pairs of primers specific to 6 E‐box elements using *35S::GFP* and *35S::MdbHLH104‐GFP* transgenic apple calli, which overexpressed *GFP* and *MdbHLH104‐GFP*, respectively. A fragment of the actin promoter containing an E‐box motif was used as a negative control. The ChIP‐PCR assay demonstrated that only the P3‐containing promoter regions of *MdAHA8*, but not the other 5 regions, were enriched by ChIP in the *35S::MdbHLH104‐GFP* transgenic calli compared to the *35S::GFP* control (Figures S3c and 2b). In addition, there are E‐box *cis* elements in the other 6 MdAHAs, that is *MdAHA1*,* MdAHA3*,* MdAHA7*,* MdAHA9*,* MdAHA11* and *MdAHA12* (Figure S3b). However, ChIP‐PCR assays demonstrated that none of them recruited MdbHLH104‐GFP proteins (Figure S3c). These results provide *in vivo* evidence for the binding of MdbHLH104 to the *MdAHA8* promoter region around the P3 element.

To verify the direct binding of MdbHLH104 to the P3‐containing recognition site in the *MdAHA8* promoter, an electrophoretic mobility shift assay (EMSA) was performed with an oligo‐probe containing a P3 cis element using purified recombinant His‐MdbHLH104 fusion protein. As a result, specific DNA–MdbHLH104 protein complexes were detected when the P3 (sequence)‐containing sequence was used as a labelled oligo‐probe. The formation of these complexes was reduced when increasing amounts of the unlabelled P3 competitor probe with the same sequence were added. This competition was not observed when the mutated version was used (Figure 2c). This specificity of competition verifies the physical interaction between the *MdAHA8* promoter region and MdbHLH104 that requires the P3 *cis* element.

To examine whether MdbHLH104 directly activates the *MdAHA8* promoter, a biochemical staining assay was performed using *GUS* as the reporter gene. The construct *P*_*MdAHA8*_ *::GUS* was genetically transformed into the WT apple calli, and then, *35S::MdbHLH104‐GFP* was introduced into the transgenic calli containing *P*_*MdAHA8*_ *::GUS* (Figure S3d). The biochemical staining assay showed that the *P*_*MdAHA8*_ *::GUS*+*35S::MdbHLH104‐GFP* double‐transformed calli have higher GUS activity than the *P*_*MdAHA8*_ *::GUS* one (Figure 2d,e), indicating that MdbHLH104 is a positive regulator for the *MdAHA8* promoter.

Taken together, it may be concluded that MdbHLH104 activates the transcription of the *MdAHA8* gene by directly binding to the P3 *cis* element in its promoter. In addition, MdbHLH104 also binds to the promoters of *MdbHLH38*,* MdbHLH39* and *MdPYE* (Figure S4a–c).

### MdbHLH104 modulates H^+^‐ATPase activity and Fe acquisition by regulating *MdAHA8* under Fe deficiency

Because it is difficult to obtain transgenic apple plants, particularly for those containing two or more exogenous genes, apple calli were thereafter used for genetic transformation and further investigation. To examine whether apple calli can be used as a model system, apple transgenic calli containing *35S::GFP* and *35S::MdbHLH104‐GFP*, respectively, were used to characterize the function of MdbHLH104 in modulating PM H^+^‐ATPase activity and Fe acquisition (Figure S5a). The result showed that MdbHLH104 increased the acidification of the apple calli and positively regulated the activity of PM H^+^‐ATPase (Figure S5b). In addition, the Fe^2+^ was analysed with a FerroZine method in the apple calli. The result showed that *35S::MdbHLH104‐GFP* transgenic apple calli exhibited a deeper colour than the *35S::GFP* control. Correspondingly, the former did accumulate more Fe^2+^ than the latter under Fe‐deficient conditions (Figure S5c). Therefore, MdbHLH104 regulated PM H^+^‐ATPase activity and Fe acquisition in the apple calli just as it did in the apple plant.

**Figure 3:**
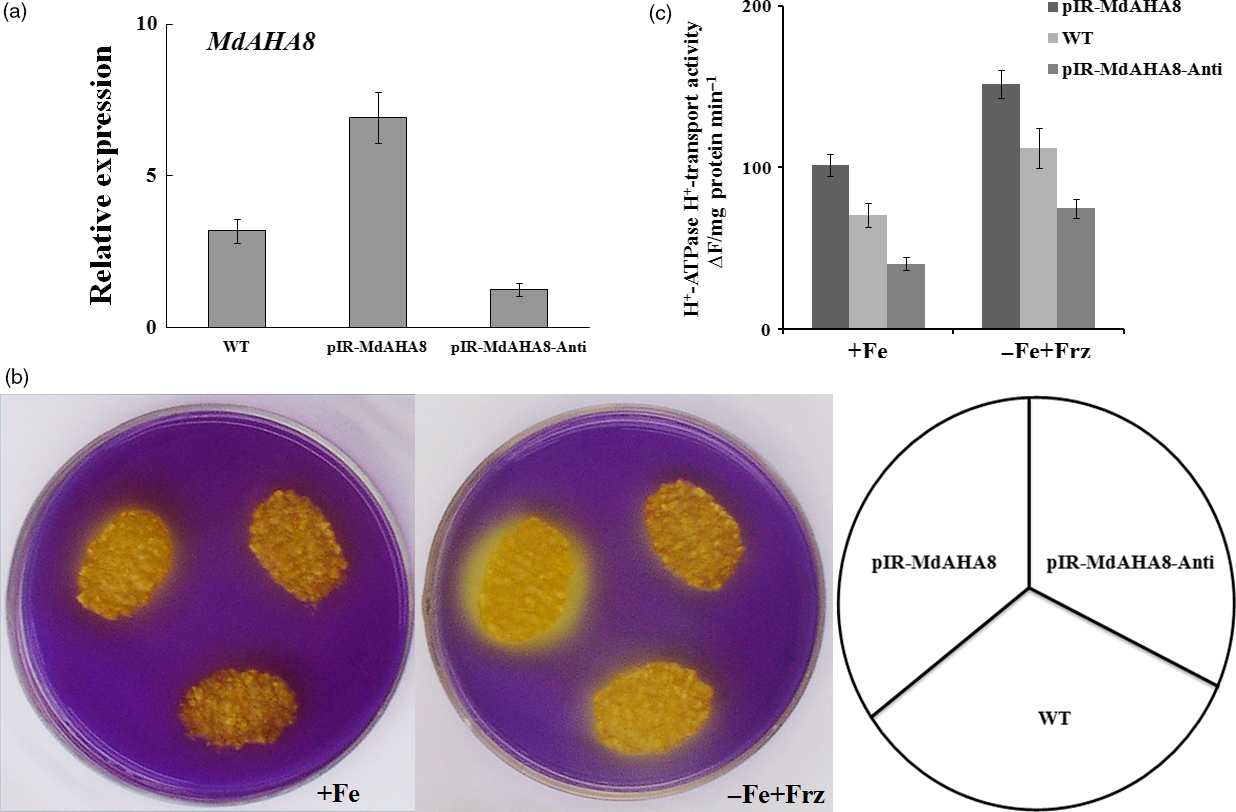
*MdAHA8* positively regulates PM H^+^‐ATPase activity. (a) The relative expression of *MdAHA8* in wild‐type, pIR‐*MdAHA8* and pIR‐*MdAHA8‐Anti* transgenic apple calli. The data represent the means ± SD of three independent experiments. (b) Acidification analysis of *MdAHA8* transgenic apple calli treated on medium containing the pH indicator dye bromocresol purple. Acidification is indicated by yellow colour around the apple calli. The same is true below unless otherwise indicated. (c) PM H^+^‐ATPase activity of wild‐type, pIR‐*MdAHA8* and pIR‐*MdAHA8‐Anti* transgenic apple calli grown for 7 days on Fe‐sufficient (+Fe) or Fe‐deficient (−Fe + Frz) media.

Using the apple calli, MdAHA8 was characterized with a function in PM H^+^‐ATPase activity and Fe acquisition. The full‐length sense ORFs and antisense cDNA fragments of *MdAHA8* were used to construct expression vectors. Two *35S*‐driven vectors, that is pIR*‐MdAHA8* for overexpression and pIR*‐MdAHA8‐Anti* for suppression, were used for genetic transformation into apple calli. RT‐PCR showed that transgenic calli pIR*‐MdAHA8* and pIR*‐MdAHA8‐Anti* were obtained and used for the determination of H^+^‐ATPase activity and Fe acquisition (Figure 3a). The result showed that the pIR*‐MdAHA8* transgenic calli exhibited higher and the pIR*‐MdAHA8‐Anti* calli exhibited lower PM H^+^‐ATPase activity than the WT control. As a result, as indicated by bromocresol purple staining into yellow colour, the pIR*‐MdAHA8* transgenic calli pumped out more and the pIR*‐MdAHA8‐Anti* calli pumped out less H^+^ into the medium than the WT control under iron‐sufficient and iron‐shortage conditions (Figure 3b). Furthermore, pIR*‐MdAHA8* transgenic calli accumulated more and the pIR*‐MdAHA8‐Anti* calli accumulated less PM H^+^‐ATPase activity than the WT control under iron‐sufficient and iron‐shortage conditions (Figure 3c). These findings indicated that MdAHA8 is involved in the regulation of PM H^+^‐ATPase activity.

**Figure 4:**
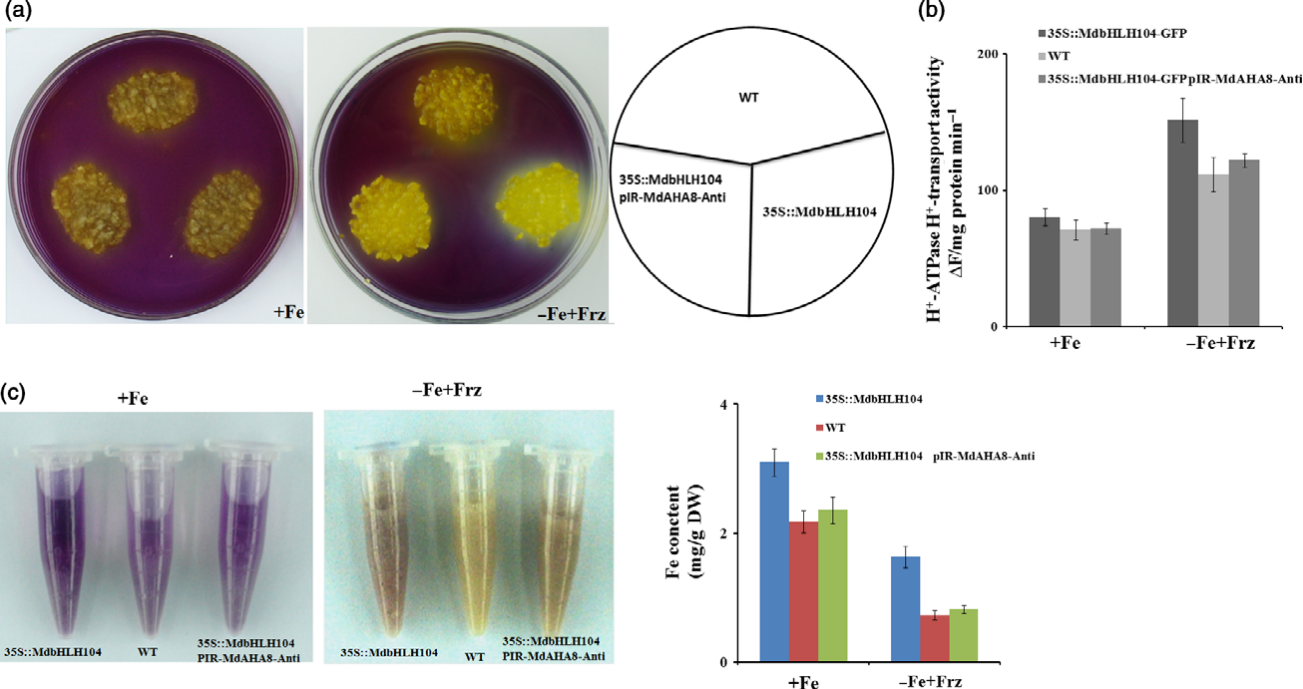
*MdAHA8* is required for MdbHLH104‐mediated acidification and iron contents of responding iron deficient. (a) Acidification of wild‐type, *35S::MdbHLH104‐GFP* and *35S::MdbHLH104‐GFP*/pIR *‐MdAHA8‐Anti* transgenic apple calli. (b) PM H^+^‐ATPase activity in vesicles isolated from wild‐type, *35S::MdbHLH104‐GFP* and *35S::MdbHLH104‐GFP*/pIR *‐MdAHA8‐Anti* transgenic apple calli treated with (+Fe) or without (−Fe + Frz) iron for 7 days. (c) Visualization of ferrous of Fe‐sufficient and Fe‐deficient conditions in wild‐type, *35S::MdbHLH104‐GFP* and *35S::MdbHLH104‐GFP*/pIR *‐MdAHA8‐Anti* transgenic apple calli by FerroZine. The resulting Fe(II) is trapped by FerroZine to produce a red product. Fe content of wild‐type, *35S::MdbHLH104‐GFP* and *35S::MdbHLH104‐GFP*/pIR *‐MdAHA8‐Anti* grown on Fe‐sufficient (+Fe) or Fe‐deficient (−Fe + Frz) media for 7 days. The data represent the means ± SD of three independent experiments. DW, dry weight.

Subsequently, the vector pIR*‐MdAHA8‐Anti* was genetically transformed into *35S::MdbHLH104‐GFP* transgenic calli. As a result, a double transgenic calli that contained *35S::MdbHLH104‐GFP* and pIR*‐MdAHA8‐Anti* were obtained and used for acidification assay and PM H^+^‐ATPase activity analysis. The result showed that the *35S::MdbHLH104‐GFP/*pIR*‐MdAHA8‐Anti* calli exhibited acidification and PM H^+^‐ATPase activity that were noticeably reduced compared with the *35S::MdbHLH104‐GFP* calli, but similar to the WT control under starvation conditions (Figure 4a,b). Furthermore, the *35S::MdbHLH104‐GFP*+pIR*‐MdAHA8‐Anti* calli accumulated less Fe than *35S::MdbHLH104‐GFP* calli, but were similar to the WT control under starvation conditions (Figure 4c). Therefore, *MdAHA8* is required for the MdbHLH104‐mediated regulation of H^+^‐ATPase activity and Fe acquisition.

### MdbHLH104 interacts with other apple IVc subgroup bHLH proteins to regulate MdAHA8 expression, PM H^+^‐ATPase activity and iron accumulation

**Figure 5:**
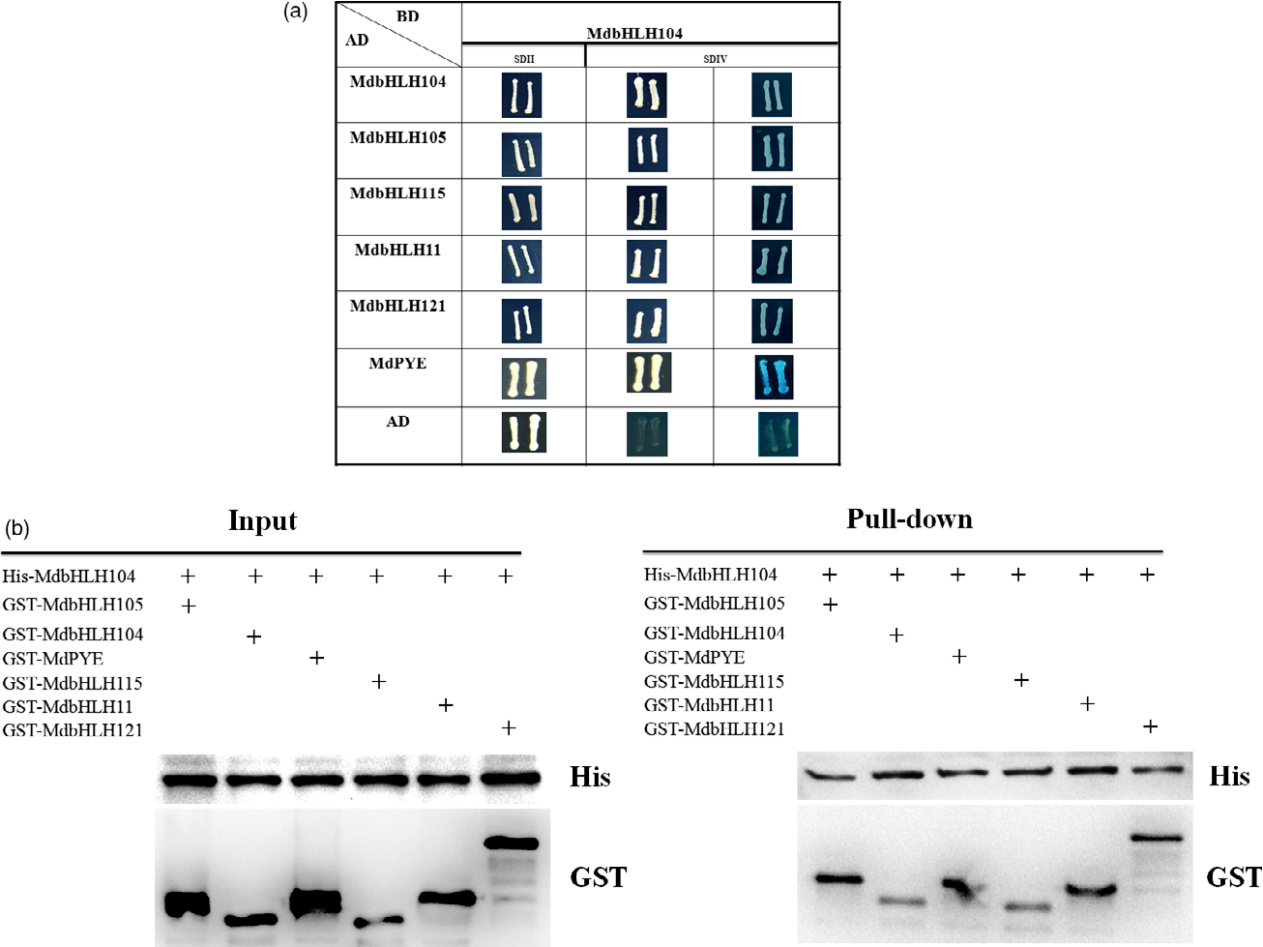
MdbHLH104 interacts with other IVc subgroup bHLH transcription factors. (a) MdbHLH104 interacts with other IVc subgroup bHLH transcription factors in yeast two‐hybrid assays. The empty vector AD plus BD‐MdbHLH104 was used as controls. The yeast cells were grown on SD/II and SDIV media. The x‐α‐gal assay was used to further confirm the positive interactions. (b) Pull‐down assay showed the interaction of His‐MdbHLH104 with GST‐MdbHLHs. The His‐MdbHLH104, GST‐MdbHLHs and GST were expressed in BL21, and then total proteins were pulled down by Ni‐agarose and detected using anti‐His and anti‐GST antibodies, respectively. ‘+’ indicates presence.

In addition to MdbHLH104, apple contains 5 other IVc subgroup bHLH TFs, that is MdbHLH105, MdbHLH115, MdPYE, MdbHLH11 and MdbHLH121. To detect whether MdbHLH104 interacts with each of them, yeast two‐hybrid assays and pull‐down analysis were conducted. The full‐length cDNA of MdbHLH104 was integrated into vector pGBT9 (BD‐MdbHLH104) as bait, whereas that of each MdbHLH105, MdbHLH115, MdPYE, MdbHLH11 and MdbHLH121 into pGAD424 (AD‐MdbHLHs) was integrated as preys. Positive X‐α‐gal activity was observed in yeasts that contained either pGBT9‐MdbHLH104 plus each pGAD424‐MdbHLHs grown on the ‐Trp/‐Leu/‐His/‐Ade screening medium, but not in those containing pGBT9‐MdbHLH104 plus the empty pGAD424 vector. The result indicated that MdbHLH104 interacted with the other IVc subgroup bHLH TFs MdbHLH105, MdbHLH115, MdPYE, MdbHLH11 and MdbHLH121 (Figure 5a). Furthermore, the interactions between MdbHLH104 and each apple IVc bHLH TFs were verified with pull‐down analysis (Figure 5b).

**Figure 6:**
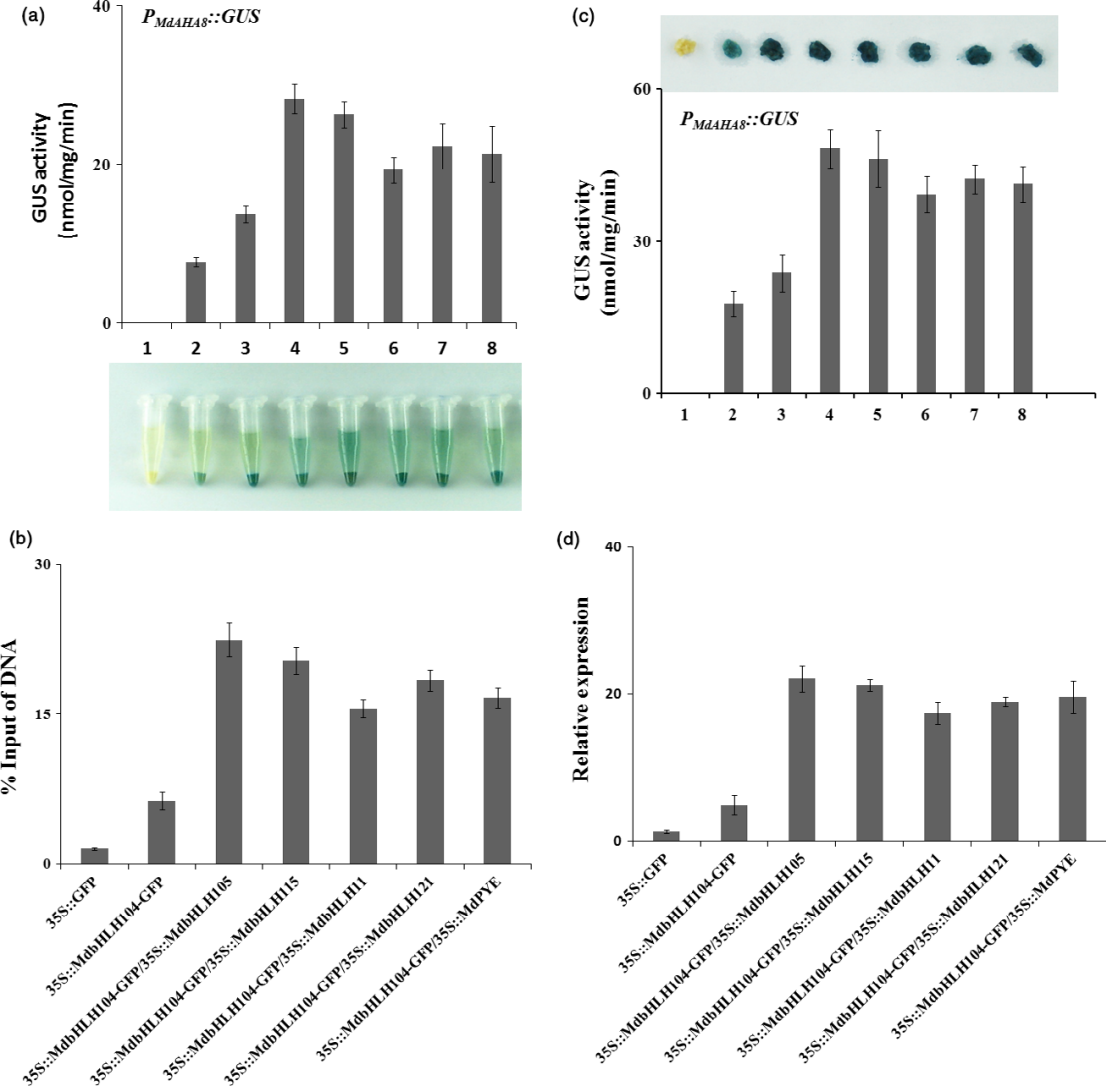
The Interaction with other MdbHLHs proteins affects the function of MdbHLH104 in the activation of *MdAHA8* promoter. (a) Transcription activation assay of *GUS* reporter gene in yeast cells. A series of transformant yeast cells containing different plasmid combinations, indicated as 1–8, were used. 1, pAD/pBD‐*GUS*; 2, pAD/pBD‐*P*_*M*__*d*_ _*AHA*__*8*_ *::GUS*; 3, pAD/pBD‐*MdbHLH104*‐*P*_*M*__*d*_ _*AHA*__*8*_ *::GUS*; 4–8, transformant yeast cells containing pBD‐*MdbHLH104*‐*P*_*M*__*d*_ _*AHA*__*8*_ *::GUS* plus pAD‐MdbHLHs plasmids, including 4, pAD‐*MdbHLH105*; 5, pAD‐*MdbHLH115*; 6, pAD‐*MdbHLH11*; 7, pAD‐*MdbHLH121*; 8, pAD‐*MdPYE*. (b) The interaction enhances the binding of the MdbHLH104 protein to the promoter fragment of the *MdAHA8* gene. The immunoprecipitated DNAs were quantified through qPCR using specific primers of candidate fragments containing the E‐box *cis* element. The results were quantified as the percentage of total input DNA by qPCR. (c) The interaction enhances the transcriptional activity of the MdbHLH104 to the *MdAHA8* promoter, as indicated by GUS staining and the activity test. The WT calli were labelled as 1; the transgenic calli, 2. After the expression vector *35S::MdbHLH104‐GFP* was cotransformed into the calli 2, transgenic calli *P*_*M*__*d*_ _*AHA*__*8*_ *::GUS*+*35S::MdbHLH104‐GFP* were obtained and labelled as 3. The transgenic calli 3 were used as the background for transient co‐expression with 5 viral vectors pIR‐*MdbHLHs* containing other IVc subgroup bHLH genes, labelled as 4–8. 4, pIR‐*MdbHLH105*; 5, pIR‐*MdbHLH115*; 6, pIR‐*MdbHLH11*; 7, pIR‐*MdbHLH121*; 8, pIR‐*MdPYE*. (d) Expression levels of *MdAHA8* gene in the WT,* 35S::MdbHLH104‐GFP* and co‐expression of bHLHs transgenic apple calli, as determined with qPCR.

Then, a yeast assay system was used to examine whether the interaction affects the function of MdbHLH104, thereby altering the activity of the *MdAHA8* gene promoter. The *MdAHA8* promoter region of 2510 bp upstream the start codon was cloned and fused with the reporter gene *GUS*, resulting in an expression cassette *P*_*MdAHA8*_ *::GUS*. The cassette was inserted into the pBD‐GAL4 vector, producing a plasmid pBD‐*P*_*MdAHA8*_ *::GUS*. Then, the coding sequence of MdbHLH104 was inserted into pBD‐*P*_*MdAHA8*_ *::GUS*, resulting in a plasmid pBD‐*MdbHLH104*‐*P*_*MdAHA8*_ *::GUS*. Meanwhile, the coding sequences of 5 other IVc subgroup bHLH TF genes were cloned into pAD‐GAL4, respectively, to generate 5 plasmids, that is pAD‐*MdbHLH115*, pAD‐*MdbHLH11*, pAD‐*MdbHLH121*, pAD‐*MdPYE* and pAD‐*MdbHLH105*. Subsequently, the pBD plasmids were genetically transformed alone or together with each pDA one into yeast cells. The histochemical assay showed the GUS activity was much higher in the transformants that contained pBD‐*MdbHLH104*‐*P*_*MdAHA8*_ *::GUS* plus each of pAD‐MdbHLHs plasmids than in those that contained it alone, indicating that the cotransformation of *MdPYE*,* MdbHLH105*,* MdbHLH115*,* MdbHLH11* and *MdbHLH121* promoted the function of MdbHLH104 to alter the activity of the *MdAHA8* promoter (Figure 6a).

To examine the biological function of the interaction between *MdbHLH104* and other IVc subgroup bHLH TFs in planta, expression vectors *35S::MdPYE*,* 35S::MdbHLH105*,* 35S::MdbHLH115*,* 35S::MdbHLH11* and *35S::MdbHLH121* were constructed and cotransformed into *35S::MdbHLH104‐GFP* transgenic calli, respectively. The resultant transgenic calli were used for ChIP‐qPCR assays with anti‐GFP antibody and primers specific to the promoter fragment of the *MdAHA8* gene. The results indicated a remarkable promotion of the recruitment of MdbHLH104 to the promoter fragment of *MdAHA8* (Figure 6b) when MdbHLH104 was cotransformed with each of the 5 other IVc subgroup bHLH TF genes.

Furthermore, a transient expression assay was conducted in transgenic apple calli to check the function of the interaction to modulate the activity of the MdAHA8 gene promoter. The coding sequences of 5 IVc subgroup bHLH TF genes were inserted into pIR viral vector, resulting in 5 transient expression viral vectors, that is pIR‐*MdPYE*, pIR‐*MdbHLH105*, pIR‐*MdbHLH115*, pIR‐*MdbHLH11* and pIR‐*MdbHLH121*. The constructs were transiently transformed into *P*_*MdAHA8*_ *::GUS* plus *35S::MdbHLH104‐GFP* transgenic apple calli background. IL60‐1 was used as a control. The results indicated that the cotransformation of each pIR‐MdbHLHs vector showed much higher GUS activity than the controls, that is IL60‐1/pIR calli and *P*_*MdAHA8*_ *::GUS* plus *35S::MdbHLH104‐GFP* (Figure 6c). Therefore, MdbHLH104 interacts with other IVc subgroup bHLH TFs to control the activity of the MdAHA8 gene.

**Figure 7:**
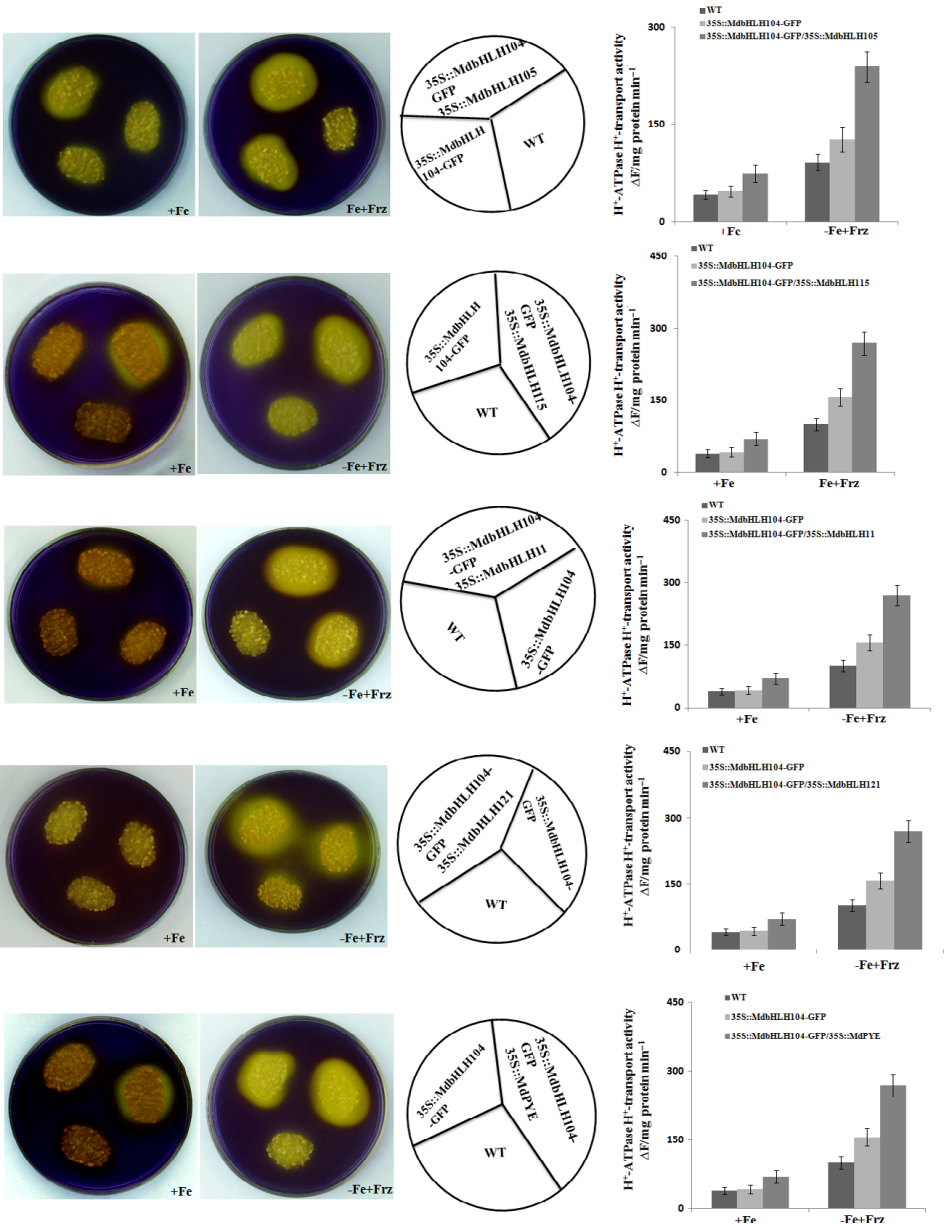
The Interaction with other MdbHLHs proteins affects the function of MdbHLH104 in the activation of PM H^+^‐ATPase. Acidification assay with the pH indicator bromocresol purple around the different apple calli as indicated (the left panels). PM H^+^‐ATPase activity in the vesicles isolated from different apple calli as indicated (the right panels).

**Figure 8:**
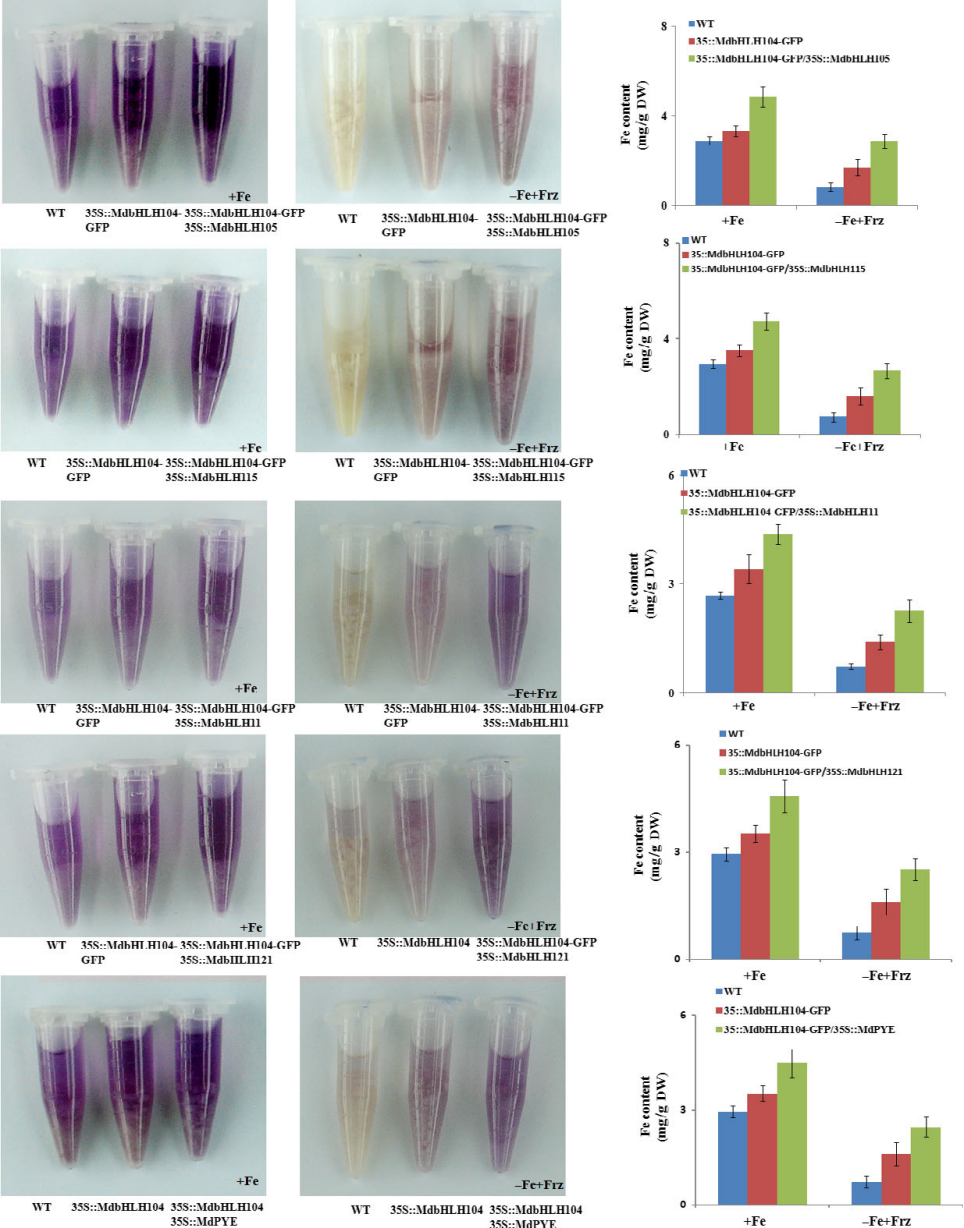
The Interaction with other MdbHLHs proteins affects the function of MdbHLH104 in iron uptake. Visualization of ferrous under Fe‐sufficient and Fe‐deficient conditions in different apple calli, as indicated. Fe contents in different apple calli, as indicated. The data represent the means ± SD of three independent experiments. DW, dry weight.

The real‐time RT‐PCR analysis showed that the co‐expression of each IVc subgroup bHLH TF together with *MdbHLH104* remarkably increased the transcript level of the *MdAHA8* gene compared to *MdbHLH104* alone (Figure 6d), demonstrating that the interaction between MdbHLH104 and each other IVc subgroup bHLH TF enhanced the expression of the MdAHA8 gene in apple calli. Finally, the PM H^+^‐ATPase activity and the iron accumulation were detected in various transgenic apple calli, including *35S::MdbHLH104‐GFP* and *35S::MdbHLH104‐GFP* plus *35S::MdPYE*,* 35S::MdbHLH105*,* 35S::MdbHLH115*,* 35S::MdbHLH11* or *35S::MdbHLH121*. The result showed that the calli that contained *35S::MdbHLH104‐GFP* plus an interacting IVc subgroup TF gene exhibited higher PM H^+^‐ATPase activity, pumped out more H^+^ into the medium and accumulated more iron than that containing *35S::MdbHLH104‐GFP* alone (Figures 7 and 8). Therefore, MdbHLH104 functions together with other apple IVc subgroup bHLH proteins to enhance PM H^+^‐ATPase activity and promote iron uptake and accumulation in transgenic apple calli.

## Discussion

Iron is essential for plants due to its various roles in life processes. Plant roots excrete protons mediated by PM H^+^‐ATPase leading to the acidification of the rhizosphere, which in turn makes iron soluble and available in soil for uptake. In addition to proton extrusion and the associated electrochemical gradient, PM H^+^‐ATPase supplies energy for iron uptake and transportation. Therefore, PM H^+^‐ATPase plays a crucial role in iron acquisition and homeostasis in plants (Guerinot and Yi, 1994; Palmgren, 2001). In *Arabidopsis*, there are 11 PM H^+^‐ATPases, which are encoded by genes *AHA1* to *AHA11* (Palmgren, 2001). Some of them are involved in Fe acquisition and homeostasis, particularly *AHA2*, which is crucial for the acidification of the rhizosphere (Baxter *et al*., 2003; Haruta *et al*., 2010). In addition, several PM H^+^‐ATPase genes have been characterized by their involvement in the response to iron deficiency and the uptake of iron elements in different plant species, such as cucumber (Santi *et al*., 2005). In this study, it was found that MdAHA8, which is one of the closest homologs among 18 apple PM H^+^‐ATPases to *Arabidopsis* AHA2, transcriptionally responds to Fe deficiency. The overexpression of *MdAHA8* promoted the proton excretion of the transgenic apple calli and increased the H^+^‐ATPase activity in response to iron deficiency (Figure 3).

Several types of TFs, such as bHLH, MYB and AP2 TFs, are involved in the regulation of Fe acquisition and homeostasis (Hindt and Guerinot, 2012; Kobayashi and Nishizawa, 2012). Among them, bHLH TFs, particularly Ib and IVc subgroups bHLH TFs, play a central role in modulating the expressions of the major Fe acquisition genes (Yuan *et al*., 2005). The first bHLH TF, which is characterized by its function in response to iron deficiency, is an Ib subgroup bHLH TF FER in tomato (Ling *et al*., 2002). FIT is its *Arabidopsis* homolog. This bHLH and other Ib subgroup bHLHs such as bHLH38 and bHLH39 (the homologs of MdbHLH38 and MdbHLH39) act together to directly regulate the expressions of *FRO2* and *IRT1* genes under Fe‐deficient and Fe‐sufficient conditions (Ivanov *et al*., 2012; Wang *et al*., 2012; Yuan *et al*., 2008). However, it seems not to directly modulate the expression of PM H^+^‐ATPase genes (Ivanov *et al*., 2012; Santi and Schmidt, 2009).

IVc subgroup bHLHs are also involved in Fe acquisition and homeostasis in *Arabidopsis*. Among them, PYE and bHLH104 regulate the acidification of rhizospheres under Fe‐deficient conditions (Long *et al*., 2010; Selote *et al*., 2015; Zhang *et al*., 2015). CmbHLH1 is a chrysanthemum homolog of bHLH105. It regulates rhizosphere acidification and Fe uptake by enhancing the transcription of an H^+^‐ATPase gene *CmHA* under iron‐starvation conditions (Zhao *et al*., 2014). However, it is unclear whether and how IVc bHLH TFs regulate the genes encoding PM H^+^‐ATPases. In this study, an IVc subgroup bHLH TF MdbHLH104 is up‐regulated by Fe starvation in apple (Figure S1). It directly binds to the promoter of *MdAHA8* and promotes its expression (Figure 2b,c), resulting in the enhanced PM H^+^‐ATPase activity (Figure 4). It is well known that PM H^+^‐ATPase plays a crucial role in proton excretion from the plant root to the rhizospheric soil and in energy‐promoted Fe uptake (Santi and Schmidt, 2009). MdbHLH104 transgenic apple plantlets and calli showed an enhanced proton excretion and an improved tolerance to Fe deficiency (Figure 1). Therefore, MdbHLH104 positively regulates the activity of PM H^+^‐ATPase and the uptake and homeostasis of iron.

In *Arabidopsis*, Ib subgroup bHLH FIT is induced and directly up‐regulates the expression of *FRO2* and *IRT1* under iron deficiency; however, its overexpression does not result in the strong induction of *FRO2* and *IRT1* under iron sufficiency (Colangelo and Guerinot, 2004; Jakoby *et al*., 2004). This is likely because these bHLH transcription factors within a subgroup form homodimer or heterodimer complexes with their family members, and both partners may be required for their regulatory function to the target genes (Heim *et al*., 2003). To be active as a transcriptional regulator, AtFIT forms heterodimers with other Ib subgroup member such as AtbHLH38, AtbHLH39, AtbHLH100 or AtbHLH101 to activate the expression of *FRO2* and *IRT1* genes (Ivanov *et al*., 2012; Wang *et al*., 2012; Yuan *et al*., 2008). Therefore, the co‐overexpression of *FIT*/*AtbHLH38*,* FIT*/*AtbHLH39*,* FIT*/*AtbHLH100* or *FIT*/*AtbHLH101* noticeably promotes iron uptake and enhances the tolerance to iron deficiency in transgenic plants (Wang *et al*., 2012; Yuan *et al*., 2008). Similarly, AtbHLH104 interacts with another IVc subgroup bHLH protein such as AtbHLH105 (ILR3), AtbHLH115 or AtPYE to form heterodimers (Selote *et al*., 2015; Zhang *et al*., 2015). In this study, it was found that MdbHLH104 interacted with another IVc subgroup member such as MdbHLH105, MdbHLH115, MdbHLH11, MdbHLH121 or MdPYE to increase the expression of the *MdAHA8* gene (Figures 5 and 6). Just like in *Arabidopsis* (Heim *et al*., 2003), the interacting partners may be required for the function of MdbHLH104 under Fe deficiency. Therefore, MdbHLH104 transgenic apple plants showed higher Fe content and ATPase activity than the WT control only under Fe‐deficient conditions, but not under Fe‐sufficient conditions (Figure 1c,d). Taken together, MdbHLH104 works together with other IVc subgroup bHLH proteins to increase the H^+^‐ATPase activity and iron content in transgenic apple calli (Figures 7 and 8). Furthermore, MdbHLH105 and other IVc subgroup bHLH members may function in a way similar to MdbHLH104, by binding to the promoter region of *MdAHA8* gene and alleviating Fe deficiency.

In addition, Ib and IVc subgroup bHLH TFs not only regulate the H^+^‐ATPase activity but also modulate other ion transports or transcription factors. ILR3 (bHLH105) plays an important role in the metal ion‐mediated auxin sensing of roots and controls metal uptake by regulating the expression of intracellular iron transport genes, such as *VIT1* (Kim *et al*., 2006; Rampey *et al*., 2006). PYE directly targets several genes such as *OPT3*,* FRD3*,* NRAMP4*,* ZIF1*,* NAS4* and *FRO3*, which are implicated in long‐distance iron transport (Long *et al*., 2010). In *Arabidopsis*, PYE directly regulates ANR1 and indirectly regulates other transcription factors, such as Ib bHLH subgroup TFs bHLH39 and bHLH101 (Long *et al*., 2010; Yuan *et al*., 2008). Additionally, bHLH104 and bHLH105 (ILR3) bind directly to the promoters of Ib subgroup bHLH genes such as *bHLH38*,* bHLH39*,* bHLH100* and *bHLH101* and to the promoter of Ib subgroup bHLH gene *POPEYE* (*PYE*; Zhang *et al*., 2015). In apple, there are four Ib and six IVc subgroup members. Among them, MdbHLH104 directly binds to the promoters of two Ib subgroup bHLH genes *MdbHLH38* and *MdbHLH39*, and to that of an IVc subgroup one *MdPYE* (Figure S4). Therefore, bHLH104 plays pivotal roles in the regulation of Fe deficiency responses via targeting *AHA* genes, Ib or IVc subgroup bHLH genes.

Iron deficiency often results in chlorosis, which affects photosynthesis and respiration (Kosegarten *et al*., 1998). Fruit trees are among the crops most affected by iron deficiency, which significantly decreases fruit yield and quality (Tagliavini *et al.,* 2001). It is well known that most fruit trees take up Fe nutrients through their rootstock. Therefore, high Fe‐efficient rootstock is a desirable trait for fruit production (Gonzalo *et al*., 2011). Numerous genes are involved in Fe response and homeostasis. However, just few of them are characterized and used for genetic improvement in fruit rootstock. In an apple rootstock *Malus xiaojinensis*,* MxFIT* is induced by iron deficiency. It ectopic expression confers improved tolerance to iron deficiency in transgenic *Arabidopsis* (Yin *et al*., 2014). Our findings regarding the regulatory mechanism involved in the iron response and homeostasis are likely to favour the development of novel biotechnological tools for the generation of rootstocks for fruit trees with the enhanced ability of nutrient‐use efficiency and adaptation to nutrient‐poor habitats.

## Materials and methods

### Plant materials and growth conditions

Tissue cultures of apple (*Malus × domestic* cv. ‘Royal Gala’) were subcultured at a 1‐month interval on an MS medium supplemented with 0.5 mg/L 6‐BA, 0.2 mg/L NAA and 0.1 mg/L GA at 25 ± 1 °C for a 16/8‐h light/night period (100 mmol/m^2^/s), whereas ‘Orin’ apple calli were subcultured at a 3‐week interval on an MS medium containing 1.5 mg/L 2,4‐D and 0.4 mg/L 6‐BA at 25 ± 1 °C under dark conditions. For Fe treatment, the calli‐grown subculture medium was an iron‐sufficient (+Fe) MS medium supplemented with 1.5 mg/L 2,4‐D and 0.4 mg/L 6‐BA for ‘Orin’ apple calli. The iron‐deficient (−Fe + Frz) medium was the same, without Fe‐EDTA and with 300 mm FerroZine, an iron indicator. For transgenic apple rooting plantlets, Hoagland's nutrient solution with or without iron was used.

### Plasmid construction and genetic transformation in apple and apple calli

The details are provided in the Data S1.

### Gene expression analysis

The details are provided in the Data S1.

### Southern blot analysis

The details are provided in the Data S1.

### Phenotypic analyses

Chlorophyll content was measured in WT and transgenic apple lines under iron‐deficient conditions. The young leaves were collected and ground into powder in liquid nitrogen. The powder was resuspended in 80% acetone and centrifuged at 10 000 ***g*** for 5 min. Chlorophyll concentrations were calculated from spectroscopy absorbance measurements at 663.2, 646.8 and 470 nm.

Acidification assays were performed as described by Yi *et al*. (1994). Wild‐type and transgenic apple calli or apple lines were grown on iron‐sufficient media for 10 days and then transferred to iron‐deficient media for 5 days. They were finally transferred to a 1% agar plate containing 0.006% bromocresol purple and 0.2 mm CaSO_4_ (pH adjusted to 6.5 with NaOH) for 24–48 h. Acidification is indicated by the yellow colour around the apple roots or calli.

FerroZine reagent forms a red‐coloured complex with ferrous, but not with ferric iron, and the Fe(II) is trapped by FerroZine to produce a red product (Stookey, 1970).

Transgenic apple lines and calli were dried for 1–2 days at 80 °C and then wet‐ashed with 1.5 mL of 13.4 m HNO_3_ and 1.5 mL of 8.8 m H_2_O_2_ for 60 min at 220 °C using a muffle furnace. Iron concentration measurement was carried out as described by Kobayashi *et al*. (2013).

### Chromatin immunoprecipitation (ChIP)‐PCR analysis

The details are provided in the Data S1.

### Electrophoretic mobility shift assay (EMSA)

*MdbHLH104* ORF was amplified with primers MdbHLH104‐F: 5′‐ATGGGGGAATGGATAGAGTAT‐3′ and MdbHLH104‐R: 5′‐AGCAGCAGGGGGCCTAAG‐3′ containing *Bam*HI and *Sal*I restriction sites, respectively. Then, the gene was inserted into the expression vector *pET32a* after digestion with *Bam*HI and *Sal*I. The resultant expression vector was transformed into *BL21*. MdbHLH104 proteins were prepared according to the instruction manual. The 36‐bp *MdAHA8* promoter probes containing a G‐box element was synthesized and labelled with biotin at the 3′ end (Sangon, Shanghai, China). Unlabelled competitor probes were generated from the dimerized oligos of the *MdAHA8* promoter regions containing E‐box motifs. The EMSA was carried out as described in the instruction manual (Thermo Scientific, Rockford). The double‐stranded oligonucleotides wt (TTYAGTYYGGGATTA***CAAATG***CAATACGGTCWTTCT) were used as probes and competitors for the EMSAs. The mut (TTYAGTYYGGGATTA*ACAAGT*CAATACGGTCWTTCT) was used as the mutated competitor.

### Protein extraction and western blotting

The details are provided in the Data S1.

### Transcription activation analysis in yeast cells

The details are provided in the Data S1.

### Transcriptional activation assays in apple calli

The details are provided in the Data S1.

### GUS analysis

The details are provided in the Data S1.

### Yeast two‐hybrid (Y2H) assay

Y2H assays were performed as described by Xie *et al*. (2012). The MdbHLH104‐coding sequence was cut with *Bam*HI and *Sal*I double digestion and cloned into pGBT9 to generate an in‐frame fusion with the GAL4 activation domain. The full‐length cDNAs of IVc subgroup *MdbHLHs* genes were cut by *Eco*RI and *Sal*I double digestion and cloned into pGAD424 to generate an in‐frame fusion with the GAL4 DNA‐binding domain. The plasmids of pGAD424‐MdbHLHs and pGBT9‐MdbHLH104 were cotransformed into yeast. The yeast clones were grown on SD/‐Trp‐Leu and SD/‐Trp‐Leu‐His‐Ade media. A selection medium supplemented with ‐Leu/‐Trp was used as a transformation control, whereas for interaction studies, ‐Leu/‐Trp/‐His/‐Ade with or without 5‐bromo‐4‐chloro‐3‐indolylb‐d‐galactopyranoside acid (x‐α‐gal) was used to test for possible interactions.

### Pull‐down assay

The assays were carried out as described by Xie *et al*. (2012). The MdbHLH104‐coding sequence was cut with *BamH*I and *Sal*I double digestion and cloned into *pET32a*, and the full‐length cDNAs of IVc subgroup MdbHLHs were cut by *EcoR*I and *Sal*I double digestion and cloned into *pGEX*. The plasmids of *pGEX*‐MdbHLHs and *pET*‐MdbHLH104 were transformed into *Escherichia coli* BL21 (DE3; Transgene, Beijing, China). For pull‐down analysis with GST‐ and His‐tagged proteins, GST‐MdbHLH105/115/11/121/PYE proteins were first eluted from glutathione–agarose beads before being incubated with His‐MdbHLH104, which that remained attached to tetradentated‐chelated nickel resin. In general, proteins were incubated at least 4 h at 4 °C under shaking conditions before being centrifuged. Precipitates were washed no fewer than three times to remove unspecific bindings and boiled (10 min, 100 °C). Then, the precipitates were further analysed by SDS–PAGE and protein gel blotting using standard procedures.

### Plasma membrane H^+^‐ATPase isolation

Transgenic apple calli and apple lines or WT controls were grown on Fe‐sufficient medium and then transformed to a Fe‐deficient medium. Plasma membranes were isolated with a buffer consisting of 15 mm Tris–Cl (pH 7.5), 0.5 m sucrose, 1 mm EGTA, 1 mm EDTA, 6% (w/v) PVP, 0.1% (w/v) BSA, 0.1 mm DTT and 1 mm PMSF. Microsomal pellets were obtained from the homogenate as described (Yang *et al*., 2010). All steps were performed at 4 °C or on ice. The homogenate was filtered through four layers of gauzes and centrifuged at 13 000 ***g*** for 10 min. The supernatant then was centrifuged for 50 min at 80 000 ***g*** to obtain a microsomal pellet that was resuspended in a buffer containing 6.4% (w/w) dextran T‐500, 6.4% (w/w) PEG 3350 (Sigma‐Aldrich, St Louis, MO), 5 mm phosphate buffer titrated to pH 7.8 with KOH, 3 mm KCl, 0.1 mm EDTA, 1 mm DTT, 1 mm PMSF, 1× protease inhibitor and 0.33 m sucrose. The final upper phases were collected, diluted with a resuspension buffer containing 0.33 m sucrose, 10% (w/v) glycerol, 0.1% (w/v) BSA, 0.1 mm EDTA, 2 mm DTT, 1× protease inhibitor and 20 mm HEPES‐KOH (PH 7.5) and centrifuged for 50 min at 100 000 ***g***. The final membrane pellets were resuspended with a resuspension buffer containing 1 mm EDTA (Yang *et al*., 2010).

### PM H^+^‐ATPase activity assays

For PM H^+^‐ATPase activity measurement, H^+^ transport activity was measured as described (Yang *et al*., 2010). An inside‐acid pH gradient (∆pH) was formed in the vesicles by the activity of the H^+^‐ATPase and measured as a decrease (quench) in the fluorescence of quinacrine (a pH‐sensitive fluorescent probe). The assays (2 mL) contained 10 μm quinacrine, 3 mm MgSO_4_, 100 mm KCl, 25 mm BTP‐Mes‐HEPES (Sigma‐Aldrich), pH 6.5, 250 mm mannitol and 50 mg/mL of a PM protein. The reactions were mixed by inversion several times and then placed in a dark chamber in a fluorescence spectrophotometer (Hitachi, Ltd., Tokyo, Japan). The reactions were initiated by the addition of ATP to a final concentration of 3 mm, and the formation of ∆pH was measured at the wavelengths of *E*_x_ = 430 nm and *E*_x_ = 500 nm. At the end of each reaction, 10 μm m‐chlorophenylhydrazone (CCCP) was added to stop any remaining pH gradient. Specific activity was calculated by dividing the change in fluorescence by the mass of PM protein in the reaction per unit time (∆*F*/min per mg of protein).

## Supporting information


**Figure S1** Alignment of apple IVc bHLH subgroup proteins and expression analysis of *MdbHLH104* gene.
**Figure S2** Construction of *MdbHLH104* overexpression vector and genetic transformation into apple plant.
**Figure S3** Identification of apple *MdAHAs* genes and ChIP‐PCR assays of MdbHLH104 protein in *MdAHA* gene promoters.
**Figure S4** MdbHLH104 protein binds to the E‐box motifs in the promoters of Ib subgroup bHLH genes *MdbHLH38* and *MdbHLH39* and in that of IVc bHLH gene *MdPYE*.
**Figure S5** Phenotypes of *35S::MdbHLH104‐GFP* transgenic apple calli under Fe‐sufficient and Fe‐deficient conditions.
**Table S1** Primers used for gene cloning.
**Table S2** Primers used for qRT‐PCR.
**Table S3** Primers used for ChIP‐PCR.
**Data S1** Supplemental materials and methods.
