# Supplementary material for: Overexpression of MdbHLH104 gene enhances the tolerance to iron deficiency in apple
Source: Plant Biotechnol J. 2016 Jan 23;14(7):1633–45. doi: 10.1111/pbi.12526 (PMC5066684; doi:10.1111/pbi.12526)
Supplement: Supplementary file 1 — Figure S1 Alignment of apple IVc bHLH subgroup proteins and expression analysis of MdbHLH104 gene. Figure S2 Construction of MdbHLH104 overexpression vector and genetic transformation into apple plant. Figure S3 Identification of apple MdAHAs genes and ChIP‐PCR assays of MdbHLH104 protein in MdAHA gene promoters. Figure S4 MdbHLH104 protein binds to the E‐box motifs in the promoters of Ib subgroup bHLH genes MdbHLH38 and MdbHLH39 and in that of IVc bHLH gene MdPYE. Figure S5 Phenotypes of 35S::MdbHLH104‐GFP transgenic apple calli under Fe‐sufficient and Fe‐deficient conditions. Table S1 Primers used for gene cloning. Table S2 Primers used for qRT‐PCR. Table S3 Primers used for ChIP‐PCR. Data S1 Supplemental materials and methods. [file PBI-14-1633-s001.docx]

**MdbHLH104** **Regulates the Activity of Plasma Membrane H^+^-ATPase and the Uptake of Iron in Apple**

Qiang Zhao, Yi-Ran Ren, Qing-Jie Wang, Yu-Xin Yao, Chun-Xiang You, Yu-Jin Hao*

National Key Laboratory of Crop Biology; National Research Center for Apple Engineering and Technology; College of Horticulture Science and Engineering, Shandong Agricultural University, Tai-An, Shandong 271018, China

**Supplemental Figure**

**
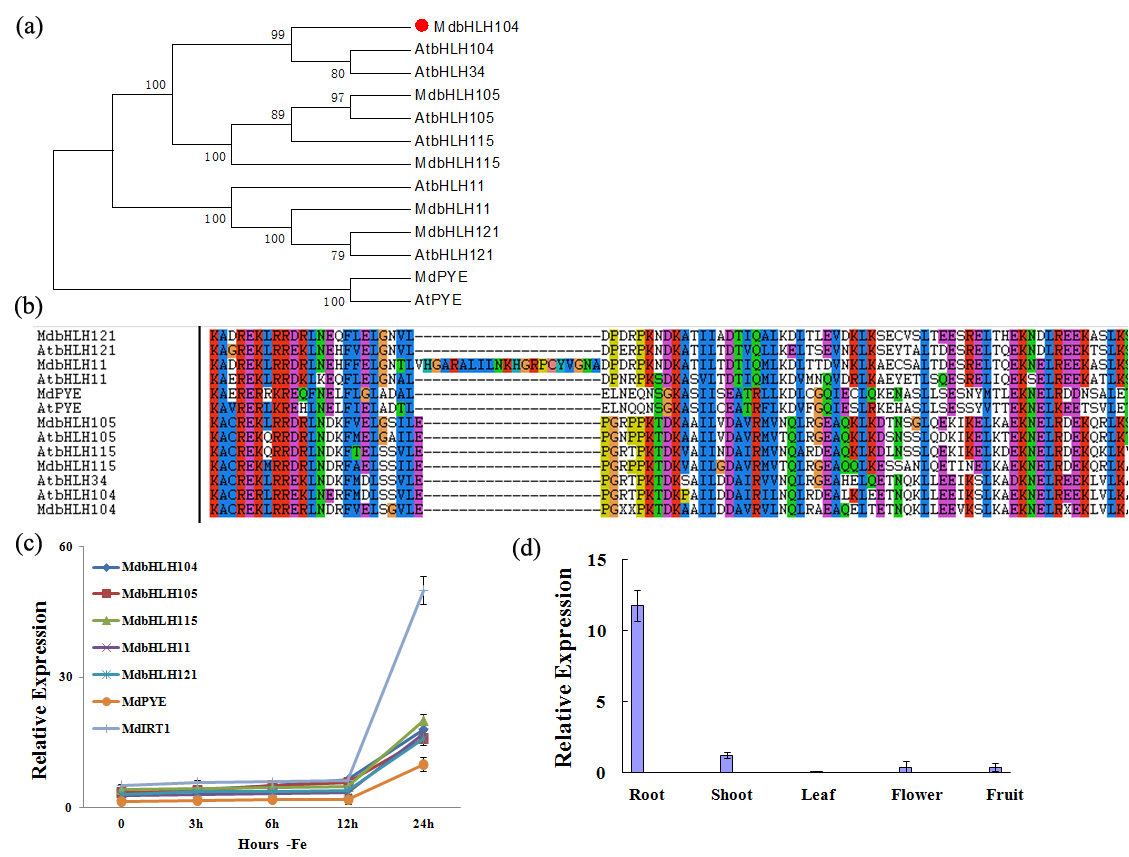
Supplemental Figure S1. Alignment of apple IVc bHLH subgroup proteins and expression analysis of *MdbHLH104* gene**

(a) Phylogenetic tree of IVc subgroup bHLH TFs in apple and *Arabidopsis.* The red point represents MdbHLH104.

(b) The bHLH domain sequences are highly conserved across all bHLH proteins in both *Arabidopsis* and apple.

(c) The expression pattern of *MdbHLH104* and other apple IVc subgroup bHLH genes in response to iron-deficient (-Fe+Frz) conditions. *MdIRT1* was used as a Fe-responsive marker gene. Apple *in vitro* shoot cultures grown under normal conditions were treated on Fe-deficient medium for 24 h and then used for RNA extraction and expression analysis.

(d) The expression levels of the *MdbHLH104* gene in the root, shoot, leaf, flower and fruit.


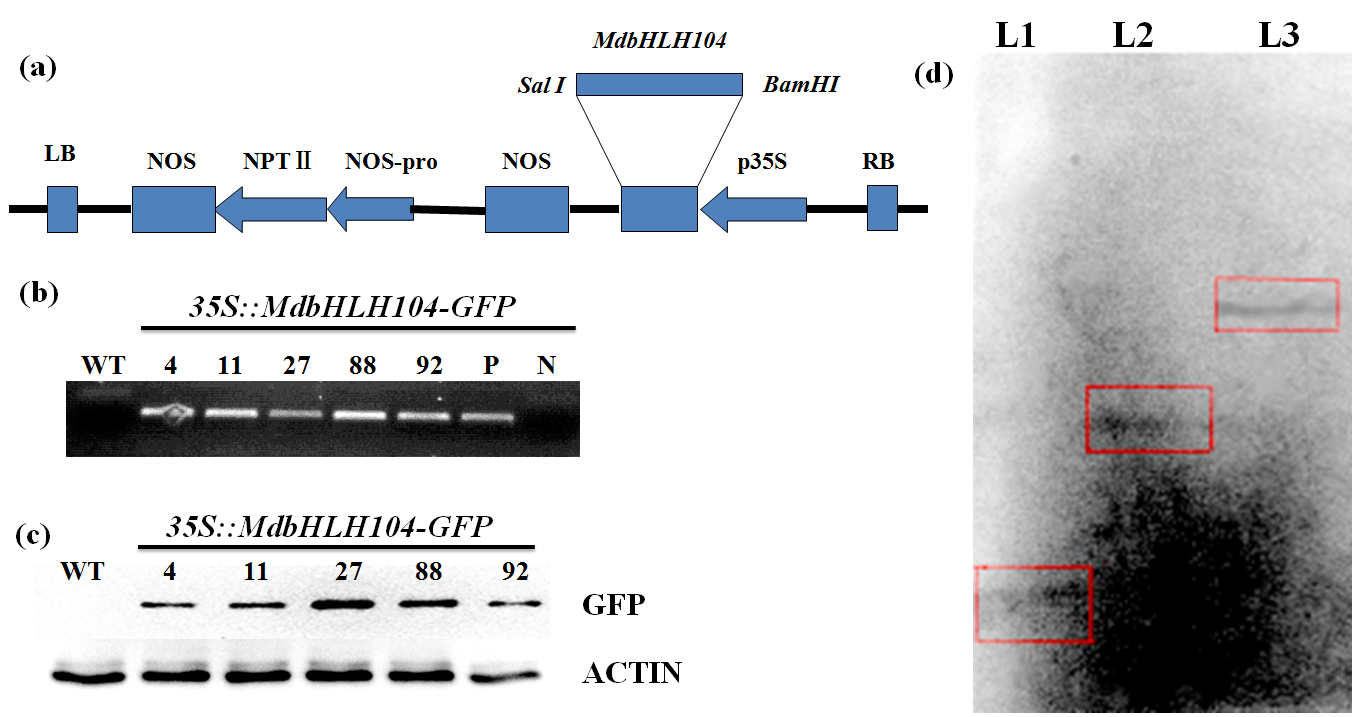
**Supplemental Figure S2. Construction of *MdbHLH104* overexpression vector and genetic transformation into apple plant**

(a) Schematic diagram of the *MdbHLH104* overexpression construct used for apple transformation. LB, left border; NOS, nopaline synthase (nos) terminator; *NPTII*, Neomycin phosphotransferase II; 35S, cauliflower mosaic virus 35S promoter; RB, right border.

(b) PCR identification of transgenic apple lines using semi-quantitative RT-PCR analysis with CaMV35S-*MdbHLH104* primers. WT, wild type; P, positive control; N, negative control. The numbers indicate different transgenic lines.

(c) The level of the MdbHLH104-GFP fusion protein in *35S::MdbHLH104-GFP* transgenic apple lines, as determined by immunoblot analysis using an anti-GFP antibody. The ACTIN was used as a loading control. The numbers indicate different transgenic lines.

(d) Southern blot analysis of transgenic apple plants. L1, L2, L3: transgenic lines.

**
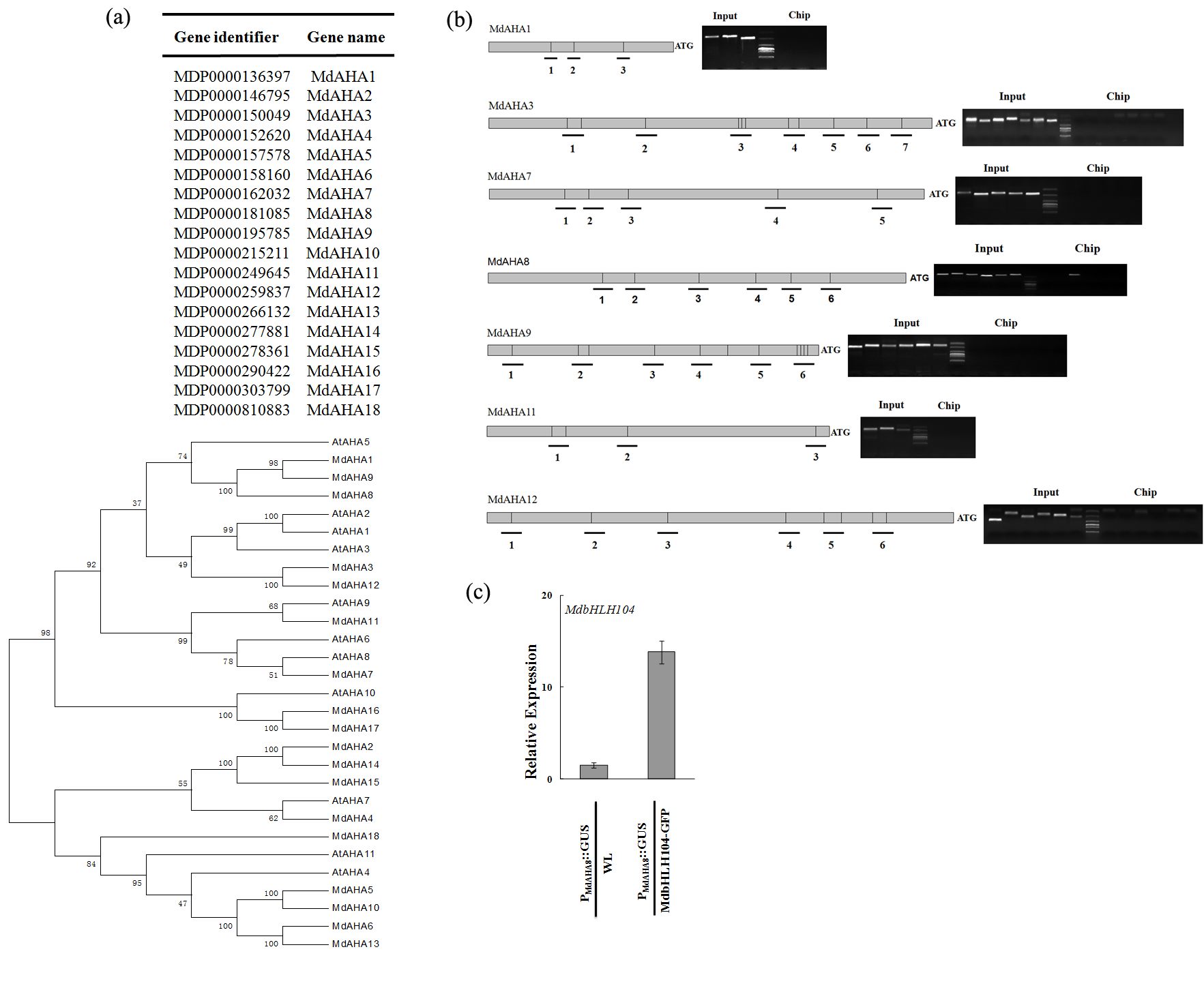
Supplemental Figure S3. Identification of apple *MdAHAs* genes and ChIP-PCR assays of MdbHLH104 protein in *MdAHA* gene promoters**

(a) The predicted AHA proteins in apple were named “MdAHA”. The predicted full-length amino acid sequences of 18 apple and 11 *Arabidopsis* AHA genes were aligned by Clustal X 1.83. The phylogenetic tree was constructed using MEGA 4.0 by the neighbor-joining (NJ) method with 1,000 bootstrap replicates.

(b) 3*5S::MdbHLH104-GFP* transgenic apple calli were used for ChIP-PCR assays. DNA was amplified using primers for the promoter regions containing the E-box (5'-CANNTG-3') *cis*-element in promoters of *MdAHAs* genes.

(c) Expression levels of *MdbHLH104* gene in *P_MdAHA8_::GUS* and *P_MdAHA8_::GUS*+*35S::MdbHLH104-GFP* transgenic apple calli.

**
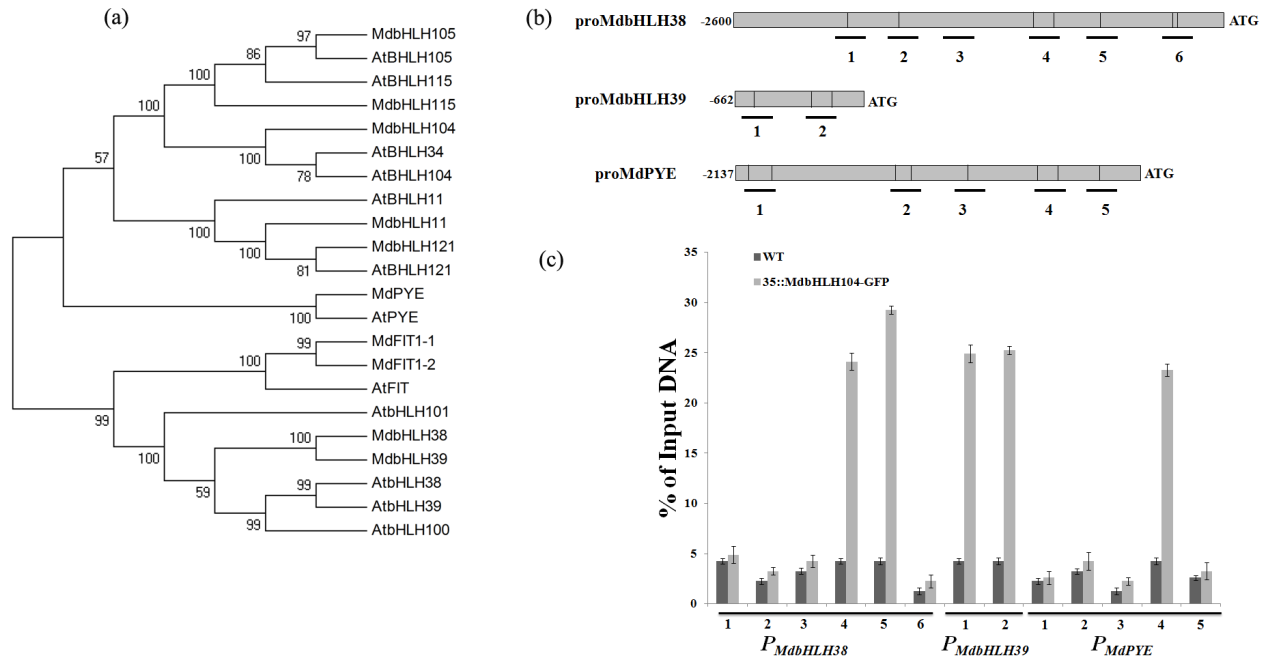
Supplemental Figure S4. MdbHLH104 protein binds to the E-Box motifs in the promoters of Ib subgroup bHLH genes *MdbHLH38* and *MdbHLH39* and in that of IVc bHLH gene *MdPYE***

(a) The phylogenetic tree of Ib and IVc subgroup bHLH transcription factors in apple and *Arabidopsis*. The phylogenetic tree was constructed using MEGA 4.0 by the neighbor-joining (NJ) method with 1,000 bootstrap replicates. Correspondingly, there are 4 Ib Subgroup bHLH genes and 6 IVc Subgroup bHLH g genes in the apple genome (<http://genomics.research.iasma.it/>).

(b) Promoters structure diagrams of *MdPYE* and Ib subgroup bHLH genes. Vertical bars show E-boxes *cis*-elements (5'-CANNTG-3'). Numbers under the boxes indicate sequences detected by ChIP-qPCR assays.

(c) ChIP-qPCR analyses of the DNA binding ratio of MdbHLH104 to the promoters of *MdPYE* and Ib subgroup bHLH genes. Chromatin from transgenic apple calli expressing *35S::GFP* or *35S::MdbHLH104-GFP* were extracted by anti-GFP antibody. qRT-PCR was used to quantify the enrichment of the *MdPYE* and Ib subgroup bHLH gene promoters. The data represent the means ± SD of three independent experiments.

**
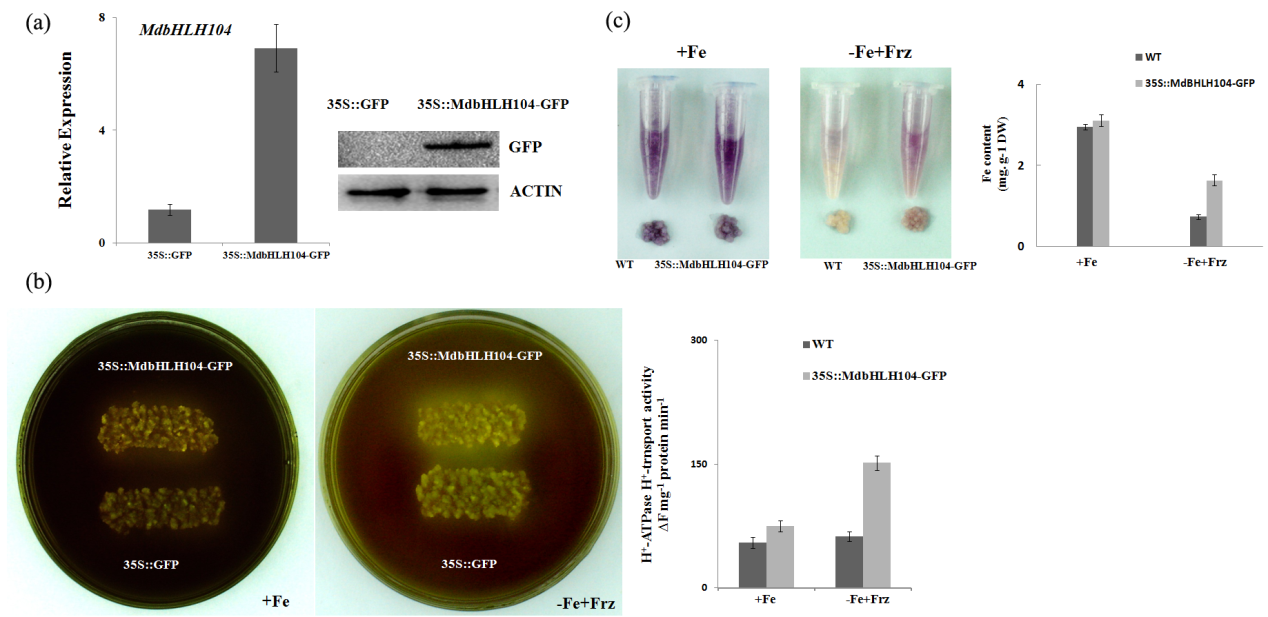
Supplemental Figure S5. Phenotypes of *35S::MdbHLH104-GFP* transgenic apple calli under Fe-sufficient and Fe-deficient conditions**

(a) Expression analysis and immunoblots assays of MdbHLH104 gene or protein in the *35S::MdbHLH104-GFP* transgenic apple calli. ACTIN was used as an internal control.

(b) Acidification of wild-type and *35S::MdbHLH104-GFP* transgenic apple calli exposed to iron-sufficient (+Fe) or iron-deficient (-Fe+Frz) media for 7 days. Acidification is indicated by the yellow color around the apple calli. The comparison of PM H^+^-ATPase activity in vesicles isolated from wild-type and *35S::MdbHLH104-GFP* transgenic apple calli treated with (+Fe) or without (-Fe+Frz) iron for 7 days.

(c) Visualization of iron and Fe content of wild-type and *35S::MdbHLH104* under Fe-sufficient (+Fe) and Fe-deficient (-Fe+Frz) conditions. The data represent the means ± SD of three independent experiments. DW: dry weight.

**Supplementary Table S1.** Primers used for gene cloning

| Gene |  | Primers |
| --- | --- | --- |
| MdbHLH11 |  | 5’ ATGGATCATTGGAAATCCG 3’ |
|  |  | 5’ GCAGTTCGATGAAGTCAGAAG3’ |
| MdbHLH104 |  | 5’ ATGGGGGAATGGATAGAGTAT 3’ |
|  |  | 5’ AGCAGCAGGGGGCCTAAG3’ |
| MdbHLH105 |  | 5’ ATGGCCTCCCCGGAAAAC3’ |
|  |  | 5’ AGCAACCGGTGGGCGGAGT3’ |
| MdbHLH115 |  | 5’ ATGGGTTCCCCGCCGCAG3’ |
|  |  | 5’ AGCAACAGGAGGGCGGAG3’ |
| MdbHLH121 |  | 5’ ATGCCCGGTGGCACCGGGCG 3’ |
|  |  | 5’ GTCGTCAGCCTTTTTGCCAC 3’ |
| MdPYE |  | 5’ ATGGGTTCACCGGATCCT3’ |
|  |  | 5’ CATATTACTAGGGTCGCTT3’ |
| MdAHA8 |  | 5’ ATGACCGTCACTGATTCCC 3’ |
|  |  | 5’ CGCTTCATCATCATCGTCA 3’ |

**Supplementary Table S2.** Primers used for qRT-PCR

| Gene |  | Primers |
| --- | --- | --- |
| MdbHLH11 |  | 5’ TCGGCTCCTTCTTCTACTCAAC3’ |
|  |  | 5’ GGGTCCTTCGTTTCTACATCAA3’ |
| MdbHLH104 |  | 5’ CCAACGACACCCCTTCTTT3’ |
|  |  | 5’ CACTCCTTTTCCTGTGACTGTG3’ |
| MdbHLH105 |  | 5’ GAAGGGACAGGCTAAATGACAA3’ |
|  |  | 5’ CTCAAACAAGAGGGAGAGAAGAG3’ |
| MdbHLH115 |  | 5’ CAATCCCAACTCCGTTTTCTG3’ |
|  |  | 5’ TGGTCCTGTGAGGTATCAACTG3’ |
| MdbHLH121 |  | 5’ TCGTGAATCTTGTGAGGTTGAG3’ |
|  |  | 5’CAAATTTGCAAAGCTCATATCG3’ |
| MdPYE |  | 5’ GCTGTCTTTGTTATGCCCTTCT3’ |
|  |  | 5’ ATGTGAGGTGGTGTTAGATGTG3’ |
| MdAHA1 |  | 5’ CCAGAGAAAACAAAAGAGAGTCAAG3’ |
|  |  | 5’ TTCACATTCACACCGAGATTG3’ |
| MdAHA3 |  | 5’ TATTCTCTGCCTGTTGGTCATC3’ |
|  |  | 5’ GCTCACTCCATTTTCCATCTCT3’ |
| MdAHA7 |  | 5’ CGCACAGCAATCACCTACAT3’ |
|  |  | 5’ TCTTCCTTGTTTCTCCCTTGAG3’ |
| MdAHA8 |  | 5’ TCGGTGTGAATGTGAAGATGAT3’ |
|  |  | 5’ TCAGGAAATACTCCAGCAAATC3’ |
| MdAHA9 |  | 5’ TTCATGGTCTTCAGCCACCT3’ |
|  |  | 5’ TCACAACTGACTCGACGTGA3’ |
| MdAHA11 |  | 5’ CTGACGAGCAGAGCCATCTT3’ |
|  |  | 5’ ATGAGAGCAAGGAGCAGAAAAC3’ |
| MdAHA12 |  | 5’ TATTCTCTGCCTGTTGGTCATC3’ |
|  |  | 5’ CGCTCCATTTTCCATCTCTAAG3’ |
| MdIRT1 |  | 5’ TTTCTGGCTTTGTGGCTATGTT3’ |
|  |  | 5’ GGCTGGAGTTTCACCATTATCT3’ |

**Supplementary Table S3.** Primers used for Chip-PCR

| Gene |  | Primers |
| --- | --- | --- |
| MdAHA1-1 |  | 5’ TCATTTCGGCGATCATCATA3’ |
|  |  | 5’ AAAGTGCCTGAAGCAGAAGC3’ |
| MdAHA1-2 |  | 5’ GTCTGTGCCTTTCCGAAATC3’ |
|  |  | 5’ AAGAATCGCCCAATAACAGG3’ |
| MdAHA1-3 |  | 5’ CGCAAGCATAATAAAAGCACA3’ |
|  |  | 5’ TCCAACAGAATGTTATTCCTTTTT3’ |
| MdAHA3-1 |  | 5’ TCGAGCATTGTTCTGATGTTG3’ |
|  |  | 5’ TAGTTGTGGCGGAGATGTCA3’ |
| MdAHA3-2 |  | 5’ CAACCCTCCTGATCCTCAAA3’ |
|  |  | 5’ AGGTGCAGTGAGCTTGGAAC3’ |
| MdAHA3-3 |  | 5’ TCATTGAGTTCCAATGACAACC3’ |
|  |  | 5’ TTGGGATCAGATTTGCTTGA3’ |
| MdAHA3-4 |  | 5’ TCCAAGGAATTGGTATGTGTGA3’ |
|  |  | 5’ TGCCTCGTTAAAACCTTGTCA3’ |
| MdAHA3-5 |  | 5’ GGGTTTTATCCTACCTTGGCTTT3’ |
|  |  | 5’ CGGTGTATCAGGTCTTCATGTG3’ |
| MdAHA3-6 |  | 5’ AACGTATGCATTTTTCCGTTT3’ |
|  |  | 5’ TTAGGACTGCAATAATCCCTCTT3’ |
| MdAHA3-7 |  | 5’ TGTAAATCTTAGAGGTTATGCATAGG3’ |
|  |  | 5’ CAGCCAAAGAGAAAAGAATGG3’ |
| MdAHA7-1 |  | 5’ ATGAGGACCGACAACACCAT3’ |
|  |  | 5’ CTGCGCTTAGCGTTGTACCT3’ |
| MdAHA7-2 |  | 5’ AGCTTGGATTTTGAGGTGGA3’ |
|  |  | 5’ TTGGTCCAACATTACTGGAAA3’ |
| MdAHA7-3 |  | 5’ TGCTGTTTTCGATATTTGTTGG3’ |
|  |  | 5’ ACCCATACCCATCACCTCAT3’ |
| MdAHA7-4 |  | 5’ GAGGAAAGTTGCACGAAGGA3’ |
|  |  | 5’ AGGAAATCACGACACCCTTG3’ |
| MdAHA7-5 |  | 5’ AACAAATAAACCCACTGGTTCAA3’ |
|  |  | 5’ ACCGAATGGACTTGGTCTTG3’ |
| MdAHA8 |  | 5’ TCAAAGATCAAGGTCTTAACCACA3’ |
|  |  | 5’ CGGTAGGTGCTCAATAGAGAATG3’ |
| MdAHA9-1 |  | 5’ ATCCTTTCCTTTTCTCTCTTGAA3’ |
|  |  | 5’ TCGAAGCGAGTGAGATGAACT3’ |
| MdAHA9-2 |  | 5’ CACCAAACTTTCAGGCCATC3’ |
|  |  | 5’ CCGAAAGTAAAAACCGAAACA3’ |
| MdAHA9-3 |  | 5’ CGTGTTTGATACGCACCTCTT3’ |
|  |  | 5’ CCCAAATCCCAAATCAAAAA3’ |
| MdAHA9-4 |  | 5’ AGTGGTATTCCTCTTTGCTTGT3’ |
|  |  | 5’ AAGAAGCAAGGGATGAGCTT3’ |
| MdAHA9-5 |  | 5’ AGAAAATTAACACAAAAGTATGTGAG3’ |
|  |  | 5’ CCAAACAAGAACACGGGTAAA3’ |
| MdAHA9-6 |  | 5’ TCATGAAGTCAACAAGGGAGAA3’ |
|  |  | 5’ TTCAGTCAACAAAAACCTCAACA3’ |
| MdAHA11-1 |  | 5’ CCTTCTAACAAAGCCGCCTA3’ |
|  |  | 5’ CCATCTCTTTGGTAGAGGTAGGTT3’ |
| MdAHA11-2 |  | 5’ AGGCAGTTCAAGTGATGTGTG3’ |
|  |  | 5’ TTAACTGCATGGACCAATCG3’ |
| MdAHA11-3 |  | 5’ TGCATGCATGAGTGCGAGT3’ |
|  |  | 5’ TACGCCAAAAACACCTCATC3’ |
| MdAHA12-1 |  | 5’ TGTACCTCAGTTGCCTTTCG3’ |
|  |  | 5’ GGGGCACAACACAACTTTTC3’ |
| MdAHA12-2 |  | 5’ AGGTTTGATTTTCGAGTCATTG3’ |
|  |  | 5’ TCATACGATATATGATAAACGGTTACG3’ |
| MdAHA12-3 |  | 5’ ACCGCTTTTACGGTTACGTG3’ |
|  |  | 5’ GGTTCAAATTAACTTCGTCAAAA3’ |
| MdAHA12-4 |  | 5’ TTCAAACTTGTTTGCCTTATATCTC3’ |
|  |  | 5’ GCCAGTTCATTTGCTTGACA3’ |
| MdAHA12-5 |  | 5’ GATGTGATTCCCCGACAGTT3’ |
|  |  | 5’ TGATGCAATTCCAGTTTTCG3’ |
| MdAHA12-6 |  | 5’ CCAAAATCATTACTTTGTCATCTCAT3’ |
|  |  | 5’ GCTTTTACTAATTTATTGTTTTGAC3’ |
|  |  |  |

**Supplemental Materials and Methods**

**Plasmid Construction, Genetic transformation in apple and apple calli**

The full-length cDNAs of different genes were amplified with primer pairs, as shown in Supplemental Table 1. Subsequently, PCR products were cloned into vector *pMD18-T* (TaKaRa, China). All genes were double-digested with *Sal*I and *Bam*HI and ligated to the *35S-GFP* vector under the control of a *CaMV 35S* promoter.

The viral vector pIR (Peretz *et al*. 2007) was used. To construct the *MdAHA8* silencing vector, the 5′-UTR of *MdAHA8* (bases 300bp) was inserted into the pIR. The resultant construct was designated as pIR-*MdAHA8-Anti*. To construct the overexpression vector, the ORFs of *MdbHLHs* and *MdAHA8* genes were inserted into the pIR vector between the *Avr*II and *Eco52*I sites, respectively. The resultant constructs were designated as pIR-*MdMdAHA8* and pIR-*MdbHLHs*, respectively. The IL-60-1 vector was used as a helper plasmid.

The *Agrobacterium tumefaciens* strain LBA4404 containing binary constructs *35S::*MdbHLH104*-GFP*, *35S::MdbHLH105*/*115*/*11*/*121*/*PYE*, *35S::GFP* and pIR-*MdbHLHs* was used to transform into apple calli, as described by Xie *et al.* (2012). ‘Orin’ apple calli were immersed into *Agrobacterium* suspension cultures for 10 min. The calli were then co-cultivated in an MS medium with 1.5 mg/L 2,4-D and 0.4 mg/L 6-BA at 25±1°C in the dark for two days. After co-cultivation, the calli were transferred to the MS screening medium containing 1.5 mg/L 2,4-D, 0.4 mg/L 6-BA, 100 mg/L kanamycin and 250 mg/L carbenicillin. The calli were subcultured at a 15-day interval for one time to obtain transgenic calli.

The apple leaves were excised from in vitro grown shoots of the cultivar one month after subculturing. Leaf strips were immersed into *Agrobacterium* suspension cultures for 10-15 min and then transferred onto an MS medium with 0.2 mg/L IAA and 2.0 mg/L TDZ for co-cultivation at 25±1°C in the dark for 2 days. Subsequently, they were shifted to an MS medium containing 0.2 mg/L IAA and 2.0 mg/L TDZ, 20 mg/L kanamycin and 250 mg/L carbenicillin regeneration and screening. After adventitious shoots were regenerated, they were transferred to an MS medium containing 0.5 mg/L 6-BA, 0.2 mg/L NAA, 0.1 mg/L GA, 20 mg/L kanamycin and 250 mg/L carbenicillin for subculturing. The plantlets were used for further investigation.

**Gene-Expression Analysis**

Total RNAs were extracted from apple calli and apple samples using the TRIzol Reagent (Invitrogen, Carlsbad, CA, USA) and the RNAplant plus Reagent (Tiangen, Beijing, China), following the manufacturer’s instructions. The first-strand cDNA was synthesized using the PrimeScript 1st Strand cDNA Synthesis Kit transcriptase (TaKaRa, Dalian, China) in accordance with the manufacturer's instructions.

qRT-PCR reactions (95°C, 7 min; 95°C, 15 s; 60°C, 1 min; 40 cycles) were performed with the SYBR green method using the iCycler iQ5 system (Bio-Rad, Hercules, CA, USA). Relative gene expression analyses were calculated by the full quantification method with *MdACTIN* as the internal control gene. At least three biological replicates were performed for each individual experiment. The primers used for qRT-PCR are shown in Supplemental Table 2.

**Southern Blot Analysis**

Total plant genomic DNA was extracted and digested with *Kpn1*. Then, the digested DNA was electrophoresed in a 0.8% (w/v) agarose gel in 1×TBE (Tris-borate-EDTA) buffers for 8 h and transferred onto the nylon membrane (Bio-Rad, Hercules, CA, USA) for 2.5 h. The probe was labeled with digoxigenin (DIG)-dUTP with DIG High Prime DNA Labeling reagents II (Roche, Mannheim, Germany), using the primers MdbHLH104-F: TTTGGAACCCGGGAGKYCGCCGAA and MdbHLH104-R: CTAAGCAGCAGGGGGCCTAAGTT. The membrane was fixed by baking at 80°C for 2h, and hybridization was carried out at 42°C for 16h. Washing, blocking and detection were carried out following the manufacturer’s instructions (Roche, Mannheim, Germany).

**Chromatin immunoprecipitation (ChIP)-PCR Analysis**

ChIP analysis was performed with the Chromatin Immunoprecipitation Assay Kit (Millipore, MA, USA) in accordance with the protocol of the manufacturer’s instructions. Protein-DNA was cross-linked for 10 min under a vacuum in a cross-link buffer, as described by Xie *et al*., (2012). Cross-linked samples were incubated in 100 mM Gly for 5 min under a vacuum, thoroughly washed in double-distilled water, and frozen in liquid nitrogen. After resuspension in lysis buffer, the purified nuclei were thenunderwent ultrasonic treatment (five times, 3 s, 40 s) to yield chromatin fragments of 300 to 500 bps. The extract was incubated with GFP antibodies (Beyotime, China). IP protein-DNA complexes were precipitated by protein A-Sepharose beads (1.5 h, 4°C).

After IP, the DNA fragments in the IP complex were released by incubating samples overnight at 65°C in an elution buffer (1% SDS, 0.1 M NaHCO_3_, and 0.25 mg/mL proteinase K). As an input control, a portion of sonicated, cross-linked and precleared DNA was treated accordingly, except for undergoing an IP. The enrichment of DNA fragments was analyzed by PCR and visualized by gel electrophoresis. Primers used for the regular PCR are shown in supplementary Table S3. The PCR reactions were incubated at 95°C for 5 min to activate the polymerase, followed by amplification at 95°C for 20 s, 60°C for 15 s and 72°C for 20 s for 30 cycles. The experiment repeated with at least three independent biological replicates. *MdACTIN* was selected as an internal control.

**Protein Extraction and Western Blotting**

A total of 2 g transgenic apple calli or apples for each sample were ground in a buffer containing 20 mM Tris (pH 7.4), 100 mM NaCl, 0.5% Nonidet P-40 (w/v), 0.5 mM EDTA, 0.5 mM PMSF and 0.5% protease inhibitor mixture (Sigma, USA). MdbHLH104 protein levels were determined by protein gel blots using an anti-GFP antibody, as described previously (Xie *et al.,* 2012). Protein extracts were separated on a 12% SDS-PAGE gel and transferred to PVDF membranes (Roche, USA) using an electrotransfer apparatus (Bio-Rad, Hercules, CA, USA). The membranes were incubated with anti-GFP (Sigma-Aldrich, St. Louis, MO, USA) or anti-ubiquitin (Sigma-Aldrich, St. Louis, MO, USA) primary antibodies and peroxidase-conjugated secondary antibodies (Abcam, Shanghai, China) before the visualization of immunoreactive proteins using ECL kits (Millipore, MA, USA). ACTIN served as a protein-loading control.

**Transcription activation analysis in yeast cells**

The promoter of *MdAHA8* with 5′-*Sac*I and 3′-*Bam*HI sites were cloned into the *pMD18-T* (TaKaRa, Dalian, China) vector from the genomic DNA of apple and then cleaved with *Sac*I/*Bam*HI and linked to the PJHA212K-GUS vector (Yoo *et al.,* 2005). The GUS expression cassettes with the *MdAHA8* promoter were cut out from the PJHA212K-GUS derivatives with *Sac*I/*Hind*III, and integrated into the pBD-*MdbHLH104* or pBD-GAL4 to generate yeast-expression plasmids pBD-GAL4-*P_MdAHA8::GUS_* and pBD-*MdbHLH104*- *P_MdAHA8::GUS_*. The plasmids were then introduced into yeast strain Y2H alone or in pairs with pAD or pAD-*MdbHLH105*/*MdbHLH115*/*MdbHLH11*/*MdbHLH121*/*MdPYE*.

**Transcriptional activation assays in apple calli**

The promoter of *MdAHA8* gene was cloned into the pXGUS-P vector (Chen *et al.,* 2009) containing the GUS reporter gene and then transformed into LBA4404. The wild-type calli was transformed through *Agrobacterium*-mediated genetic transformation, and *P_MdAHA8_::GUS* transgenic calli was identified with GUS Staining. The ORFs of IVc subgroup *MdbHLHs* genes were inserted into the pIR viral vector as described by Peretz et al. (2007). IL60-1 was used as a helper plasmid. The plasmids were transformed into the *P_MdAHA8_::GUS*+*35S::MdbHLH104-GFP* calli.

**GUS analysis**

For histochemical staining, the transgenic apple calli or yeast cells were immersed in GUS staining buffer (1 mM 5-bromo-4-chloro-3-indolyl-β-glucuronic acid solution in 100 mM sodium phosphate pH 7.0, 0.1 mM EDTA, 0.5 mM ferrocyanide, 0.5 mM ferricyanide, and 0.1% Triton X-100) at 37°C for 1 h. After staining, the transgenic apple calli or yeast cells were photographed.

For the quantitative analysis of GUS activity, the proteins were extracted with 1 mL of extraction buffer (50 mM NaHPO_4_ PH 7.0, 10 mM β-mercaptoethanol, 10 mM Na_2_EDTA, 0.1% Triton X-100) and 1 mL RIPA Lysis Buffer (Beyotime, China) from the transgenic apple calli and yeast cells. The concentration of total protein was determined with the Protein Assay kit (Bio-Rad, USA). The extract 100 µL was then added to 900 µL of GUS reaction buffer containing 1 mM 4-methylumbelliferone glucuronide (4-MUG), and the mixture was incubated at 37°C. After the reaction proceeded for 0, 5, 10, 15, 30, and 60 min, 100 µL of the reaction mixture was added to 900 µL of the stop solution (1 M sodium carbonate). The fluorescence was measured using a VersaFlour spectrofluorometer at an excitation wavelength of 365 nm and an emission wavelength of 450 nm.
